# Overcoming the Low‐Temperature Barrier: Controlling Li₂S Deposition and Enhancing Catalysis in Lithium‐Sulfur Batteries Using Island‐like Bi₂O₃ on rGO

**DOI:** 10.1002/advs.202502045

**Published:** 2025-03-17

**Authors:** Hai‐Ji Xiong, Ding‐Rong Deng, Yu‐Lin Luo, Jia‐Xi Song, Jin‐Wei Yan, Shuang‐Lin Cai, Jia Liang, Cheng‐Wei Zhu, Ye Zeng, Gui‐Fang Li, Yi Li, Wen‐Jun Zhang, Mei‐Lin Liu, Qi‐Hui Wu

**Affiliations:** ^1^ College of Marine Equipment and Mechanical Engineering Key Laboratory of Energy Cleaning Utilization Development Cleaning Combustion and Energy Utilization Research Center of Fujian Province Xiamen Key Laboratory of Marine Corrosion and Smart Protective Materials Jimei University Xiamen Fujian 361021 P. R. China; ^2^ Jiangsu Key Lab of Advanced Functional Polymer Design and Application Department of Polymer Science and Engineering College of Chemistry Chemical Engineering and Materials Science Soochow University Suzhou 215123 P. R. China; ^3^ City Univ Hong Kong Dept Mat Sci & Engn Ctr Super diamond & Adv Films COSDAF 83 Tat Chee Ave Hong Kong 999077 P. R. China; ^4^ School of Materials Science & Engineering Center for Innovative Fuel Cell and Battery Technologies Georgia Institute of Technology Georgia 30332 USA

**Keywords:** island‐structured, Li_2_s deposition, lithium‐sulfur batteries, low temperature, rate‐determinate step

## Abstract

Low‐temperature lithium‐sulfur batteries (LSBs) face challenges such as Li₂S accumulation and the slow conversion of lithium polysulfides (LiPSs), significantly affecting their capacity and cycling life. While functionalizing cathode shows potential to overcome these problems, there has been little focus on understanding the deposition behavior of Li₂S at low temperatures and the specific catalysis processes of newly identified platforms. Here we report an island‐like Bi₂O₃ uniformly distributed on reduced graphene oxide (IBG) as a sulfur host material. This unique island‐like structure increases the contact area between the electrolyte and electrode, thus enhancing Li⁺ transport efficiency. More importantly, the IBG structure exhibits a targeted catalytic ability toward LiPSs at low temperatures, significantly accelerating the conversion of Li₂S₈ to Li₂S₄ and subsequently to Li₂S. Moreover, the nucleation of Li₂S on the IBG cathode follows a progressive mode with fewer nuclei, effectively preventing Li₂S accumulation and enhancing the battery's charge–discharge efficiency. As a result, LSBs using IBG as the sulfur host can operate reliably at extremely low temperatures, down to −60 °C. This remarkable performance broadens the operating temperature range of LSBs and offers valuable insights for selecting high‐performance cathode modification materials in the future.

## Graphical abstract

A new island‐like Bi₂O₃ uniformly distributed on the reduced graphene oxide (IBG) is synthesized as a sulfur host material. IBG's special targeted catalytic ability toward two rate‐determining steps at low temperatures. IBG enhances catalytic efficiency at low temperatures, preventing Li₂S accumulation and improving charge–discharge efficiency. Consequently, lithium‐sulfur batteries with IBG as the sulfur host can operate at −60 °C.

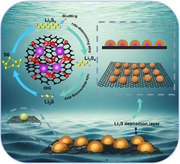

## 1 Introduction

Lithium‐sulfur batteries (LSBs) are an emerging type of secondary battery that utilizes sulfur (S₈) as the cathode material and metallic lithium (Li) as the anode. Their operation mainly involves the multi‐step reduction of sulfur and the formation and conversion of lithium polysulfides (LiPSs).^[^ ^1^, ^2^, ^3^ ^]^ Compared to traditional lithium‐ion batteries, LSBs are considered one of the most promising energy storage systems due to their high theoretical energy density (2600 Wh kg⁻¹),^[^ ^4^ ^]^ and the abundance, low cost, and non‐toxicity of sulfur. They hold great appeal for applications in electric vehicles, portable electronic devices, and grid‐scale energy storage.^[^ ^5^, ^6^, ^7^ ^]^ However, their development still faces several challenges, including the poor conductivity of sulfur, the “shuttle effect” of LiPSs, and the volume expansion of electrodes during charge–discharge cycling.^[^ ^8^, ^9^, ^10^, ^11^, ^12^ ^]^ Over the past decades, various strategies have been proposed to address these challenges, such as separator modification, binder selection, electrolyte optimization, and anode protection. These approaches have improved the performance of LSBs to some extent. In particular, cathode functionalization offers distinct advantages over other strategies by acting directly on the sulfur cathode, enhancing charge transfer and reaction kinetics of active materials, and significantly suppressing the LiPSs shuttling.^[^ ^13^, ^14^, ^15^, ^16^, ^17^, ^18^, ^19^, ^20^ ^]^ These benefits make it a standout approach for enhancing the energy density and cycling stability of LSBs. Additionally, cathode functionalization is flexible in design, cost‐effective, and compatible with other optimization strategies, establishing it as one of the core technologies for enhancing LSB performance.^[^ ^21^ ^]^ Unfortunately, most high‐performance LSB strategies have been developed for room‐temperature operation, with very few studies addressing performance at extremely low temperatures.

However, humanity's gradual understanding of the world has been marked by the exploration of extreme environments, including polar regions, the deep sea, and outer space. These environments are often highly complex and variable, posing critical challenges for energy systems to function reliably at extremely low temperatures. As a key component of energy systems, the performance of batteries plays a decisive role in ensuring the operation of devices. LSBs face significant challenges in maintaining normal operation under low‐temperature conditions. For instance, the solubility of dissolved polysulfides in the electrolyte typically decreases at low temperatures. Due to the reduced solubility, the conversion reactions of sulfides become incomplete, leading to a decrease in discharge capacity and potentially causing sulfide deposition, which negatively impacts the reversibility and cycling stability of the battery. Additionally, insufficient sulfide solubility may lead to the accumulation of sulfides on the lithium anode, exacerbating the shuttle effect. At low temperatures, the molecular motion of solvents and solutes in the electrolyte slows down, and the interactions between solvent molecules strengthen, resulting in reduced fluidity, which significantly impairs the migration and diffusion rates of lithium ions. The ionic conductivity of the electrolyte also decreases significantly as the temperature drops, and the dissociation of lithium salts in the electrolyte may also decrease at low temperatures. The decrease in ionic conductivity directly leads to an increase in the internal resistance of the battery, making the charging process more difficult and reducing the charging and discharging efficiency of the battery. The traditional lithium‐ion batteries, which operate based on the “rocking chair” mechanism, experience a significant decline in discharge capacity, dropping below 40% of room temperature capacity when the temperature falls below −20 °C^[^ ^22^ ^]^ LSBs, relying on a multi‐electron reaction mechanism with multi‐phase conversion processes, are even more severely affected by low temperatures compared to traditional lithium‐ion batteries.^[^ ^23^, ^24^, ^25^, ^26^ ^]^ For example, Diao et al. recently developed a molybdenum thiophosphate [Mo₂S₁₂]^2^⁻ cluster as a sulfur host material, effectively limiting LiPSs shuttling. While this material achieves a reversible specific capacity of ≈1100 mAh g⁻¹ at room temperature and a current density of 1C, its capacity drops sharply to less than 300 mAh g⁻¹ at −20 °C.^[^ ^27^ ^]^

At normal temperatures, the discharge process of LSBs typically exhibits two distinct plateaus in electrochemical tests. The first plateau, occurring ≈2.3 V (vs Li⁺/Li), corresponds to the reduction of S₈ to high‐order soluble LiPSs (e.g., Li₂S₈), which are further reduced to Li₂S₄. During this stage, the high solubility of LiPSs leads to their diffusion into the electrolyte, causing the “shuttle effect”, which decreases Coulombic efficiency and contributes to capacity decay. The second plateau typically observed at ≈2.1 V (vs Li⁺/Li), corresponds to the reduction of soluble LiPSs (e.g., Li₂S₄) to solid low‐order sulfides (e.g., Li₂S₂ and Li₂S). This stage represents the solid‐phase reaction of sulfur conversion and usually accounts for 70% to 80% of the total capacity, making is the main contributor to the overall capacity of LSBs.^[^ ^28^, ^29^, ^30^ ^]^ The reaction kinetics at this stage are relatively slow, especially the conversion of Li₂S₂ to Li₂S. Due to the low conductivity of Li₂S, this process can induce significant polarization, limiting the battery's rate performance. Therefore, the general approach to improving the performance of LSBs at normal temperatures is to enhance the adsorption capacity of LiPSs and accelerate the sulfur conversion reaction rate.^[^ ^31^, ^32^, ^33^, ^34^ ^]^ At low temperatures, as demonstrated in our previous research,^[^ ^35^ ^]^ LSBs exhibit three discharge plateaus, corresponding to the transition from S₈ to Li₂S₈, Li₂S₈ to Li₂S₄, and further solid‐phase stages. Further research has shown that the conversions from Li₂S₈ to Li₂S₄ and from Li₂S₄ to Li₂S₂/Li₂S are the two rate‐determining steps for LSBs at low temperatures. Most current research on LSBs at low temperatures mainly focuses on the adsorption‐catalysis‐conversion of LiPSs. For example, Zheng et al. reported a thick, independent TiO_2_ nanoparticle‐embedded 3D carbon composite (TiO₂@C@CSC) host with highly oriented channels. These channels facilitated ion/electron transport, while the TiO₂@C nanoparticles enhanced the adsorption and conversion of LiPSs, achieving a capacity release of ≈200 mAh g⁻¹ at −40 °C.^[^ ^36^ ^]^ Since the conversion of LiPSs to solid precipitates is the critical rate‐determining step, where lithium ions need to diffuse through the electrolyte to the electrode interface and react with the sulfides to form Li₂S. At low temperatures, the diffusion rate of ions slows down, preventing the efficient deposition of lithium ions as Li₂S on the anode surface, which results in a decrease in the battery's capacity. Therefore, guiding the deposition behavior of Li₂S is particularly important for improving the performance of low‐temperature LSBs. However, previous research has largely overlooked the deposition behavior of Li₂S at low temperatures.

**Scheme 1:**
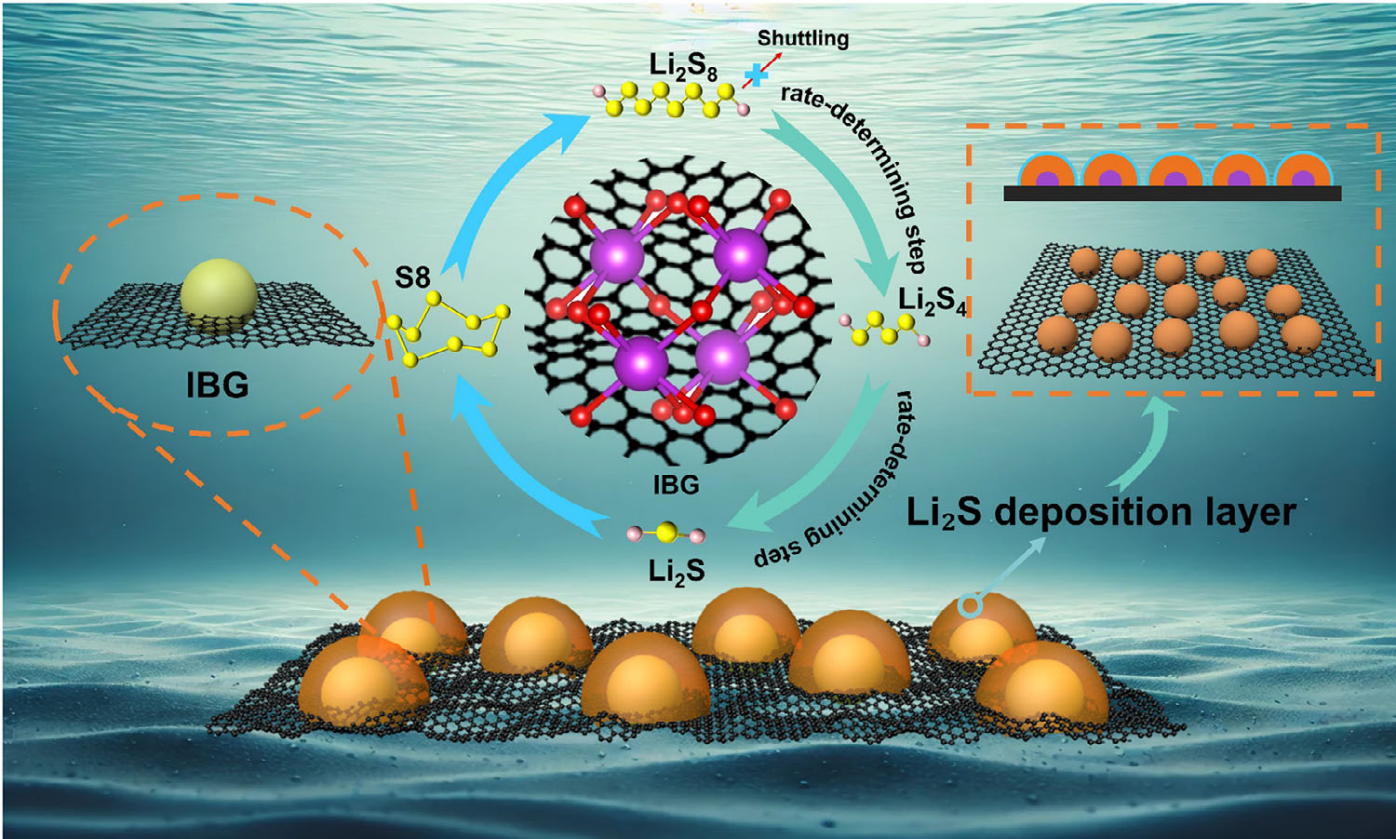
Schematic illustration of island‐like Bi_2_O_3_ distributed on reduced graphene oxide, a schematic of the conversion steps of LiPSs, and a schematic of the deposition morphology of Li_2_S at low temperatures.

In response to the above issues, Considering that Bi₂O₃ and other bismuth (Bi)‐based compounds have been proven to be effective photocatalysts and electrocatalysts.^[^ ^37^, ^38^ ^]^ Additionally, Bi₂O₃ has been reported to exhibit a strong affinity for LiPSs.^[^ ^39^ ^]^ Bismuth is a relatively rare element, but it is more environmentally friendly than some rare earth metals, and its extraction and processing have relatively minor environmental impacts. However, the potential of these bismuth‐based catalysts in LSBs has not been fully realized due to their insufficient electronic conductivity. Reduced graphene oxide (rGO) is a material with exceptional electrical conductivity, capable of significantly enhancing the electronic conductivity of electrodes. As a carbon‐based material, rGO is theoretically abundant in natural resources and can be synthesized and recycled through various methods, offering good sustainability. Furthermore, the production processes of graphene materials are continuously improving, promoting their commercialization and sustainability. Exploiting the diffusion characteristics of bismuth during high‐temperature calcination in carbon materials, this study investigates an island‐like composite material (IBG) synthesized through the in situ embedding of microspherical Bi₂O₃ into rGO, and examine its properties as a sulfur host material for LSBs. At room temperature, adsorption experiments of Li₂S₆, UV–vis spectroscopy, and adsorption energy calculations indicate that IBG exhibits strong adsorption of soluble LiPSs. The unique island‐like structure also helps shorten ion transport pathways, enhancing performance. Additionally, IBG effectively lowers the reaction energy barriers, facilitating the reaction process. This promotes the smooth capture, diffusion, and conversion of LiPSs during the charge‐discharge cycle, helping to mitigate the negative effects of the “shuttle effect” (e.g., side reactions with anode materials and increased electrochemical polarization), thereby enhancing the cycling stability of LSBs. At low temperatures, IBG maintains the advantages observed at room temperature, further improving performance. More importantly, compared to the solid‐phase reaction process of Li₂S₄ to Li₂S₂/Li₂S, which determines low‐temperature performance, IBG also exhibits specific catalytic effects on another rate‐determining step, the reduction of Li₂S₈ to Li₂S₄. By observing the morphology of Li₂S deposition and comparing it with theoretical nucleation models, it is found that IBG effectively inhibits the accumulation and growth of Li₂S (as shown in **Scheme** **1**). This deposition method promotes the formation of a large electrode/electrolyte contact interface, enhancing both electron and ion transport, which in turn improves the rate of electrochemical reactions. Results show that LSBs with IBG as the sulfur host exhibit a discharge capacity of 597 mAh g⁻¹ at room temperature and maintain a high current density of 5C. At 2C current density, the reversible capacity reaches 745 mAh g⁻¹ after 1000 cycles, with a capacity decay of only 0.016% per cycle. When the temperature drops to −20 °C, a capacity of ≈900 mAh g⁻¹ is still achieved at a current density of 0.1C. Due to the special deposition form, the oxidation reaction of Li₂S dissolution is promoted, leading to a Coulombic efficiency of ≈100%. Even at −50 °C, the battery continues to undergo solid‐phase reactions. More importantly, it can still operate normally at −60 °C, delivering an initial discharge capacity of ≈400 mAh g⁻¹. This is the first report of LSBs functioning normally at such low temperatures with significant capacity.

## 2 Results and Discussion

**Figure 1:**
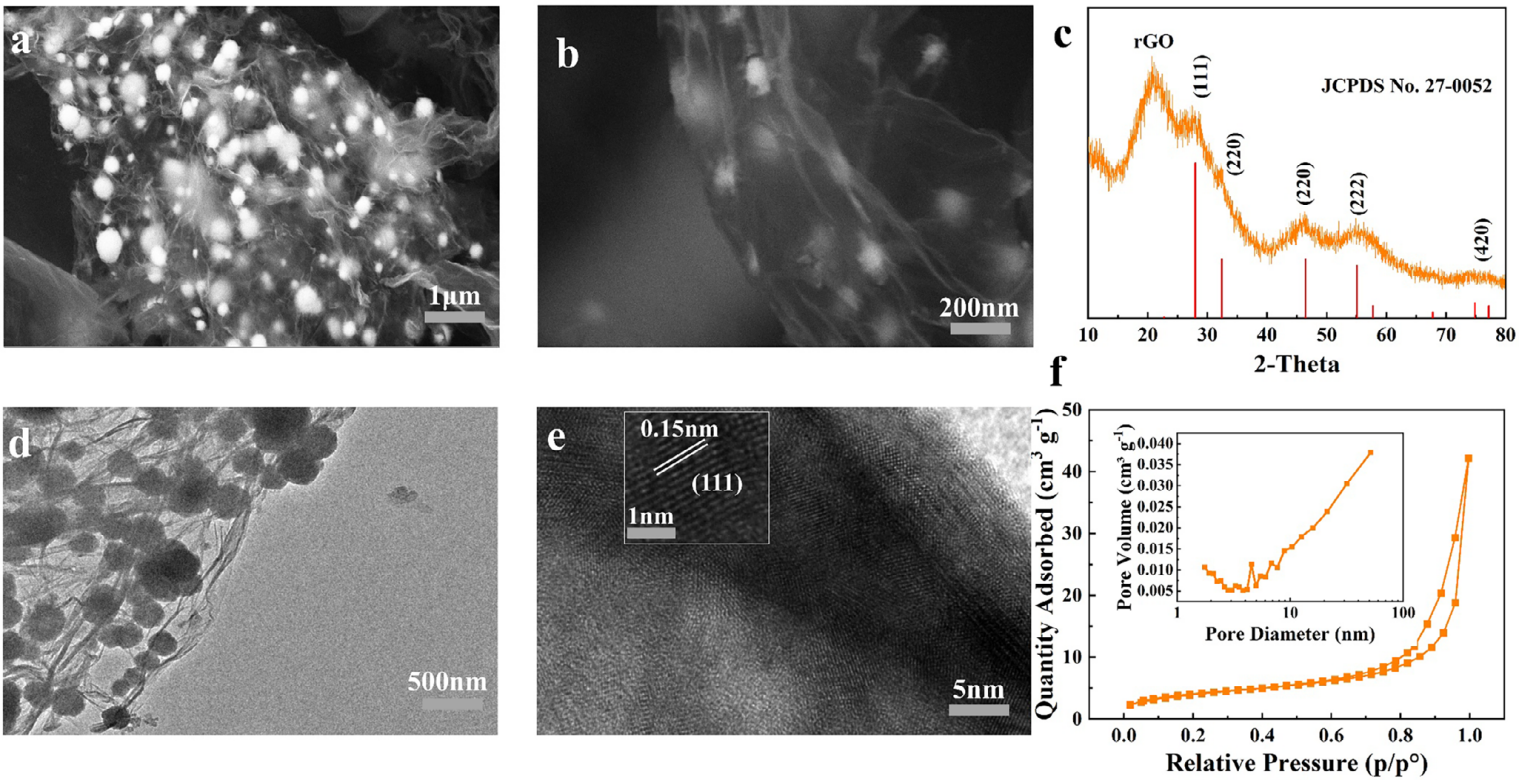
Morphology and microstructure characterizations of the IBG sample. a) and b) SEM images, c) TEM image, and d) HRTEM image of the IBG sample, e) XRD pattern, and f) N_2_ adsorption‐desorption isotherms and pore size distribution (inset) of IBG.

The morphology and microstructure of the prepared IBG were characterized using scanning electron microscopy (SEM) and transmission electron microscopy (TEM). As shown in **Figure** **1**a, the prepared sample is composed of microspherical particles with a diameter of ≈200 nm, and no significant agglomeration is observed. From the magnified image in Figure 1b, it is evident that the microspherical Bi₂O₃ particles are embedded on the rGO surface, forming an island‐like structure with a highly uniform distribution. The layered structure of rGO provides robust support for these particles, enhancing the conductivity and surface activity of the material. This structural design contributes to improved electrochemical performance in LSBs, as demonstrated in subsequent studies. Bi₂O₃ particles without rGO tend to aggregate, forming micron‐sized particles (Figure S1, Supporting Information). The layered structure of rGO effectively isolates the Bi₂O₃ nanoparticles, preventing their aggregation during the reaction process. This allows the nanoparticles to be uniformly distributed on the rGO surface, minimizing direct particle‐to‐particle contact and preventing agglomeration. The rGO content in IBG was determined using thermogravimetric analysis (TGA) and was calculated to be ≈9.8% (Figure S2, Supporting Information). TEM images reveal the specific distribution of Bi₂O₃ nanoparticles embedded within the rGO layers (Figure 1c), showing good contact between the particles and the rGO. High‐resolution TEM (HRTEM) images show distinct lattice fringes (Figure 1d) with a lattice spacing of 0.15 nm, corresponding to the (111) crystal plane of Bi₂O₃. This observation confirms the high crystallinity and effective isolation of Bi₂O₃ in the composite material, which enhances charge transfer and facilitates the full utilization of active materials in LSBs. The X‐ray diffraciton (XRD) patterns (Figure 1e) show that the main peaks match the JCPDS card No. 27–0052 (Bi₂O₃), confirming the presence of well‐crystallized Bi₂O₃ in IBG. The peak positions correspond to the characteristic diffraction peaks of Bi₂O₃, such as (111), (220), and (222), highlighting its crystalline structure. Additionally, the XRD pattern of Bi₂O₃ in Figure S3 (Supporting Information) also matches the main peaks of the JCPDS card No. 27–0052, further corroborating the crystalline nature of Bi₂O₃.

The pore distribution and specific surface area of IBG were analyzed using N₂ adsorption‐desorption isotherms. As shown in Figure 1f, the IBG sample exhibits a large specific surface area of 129.3 m^2^ g⁻¹ and a high pore volume of 0.46 cm^3^ g⁻¹. In comparison, Figure S4 (Supporting Information) shows that Bi₂O₃ alone has a specific surface area of 43.78 m^2^ g⁻¹ and a pore volume of 0.05 cm^3^ g⁻¹. These results indicate that the incorporation of rGO significantly increases the specific surface area and pore volume of IBG. The adsorption isotherms of IBG exhibit typical type IV characteristics, with a notable increase in adsorption at higher relative pressures, signifying the presence of abundant mesoporous structures in the material. The pore size distribution, shown in the inset of Figure 1f, indicates that the pore sizes are mainly in the 2–10 nm range. This mesoporous structure improves the electrolyte permeability and the utilization efficiency of active materials, thereby enhancing the rate performance and cycling stability of LSBs.

**Figure 2:**
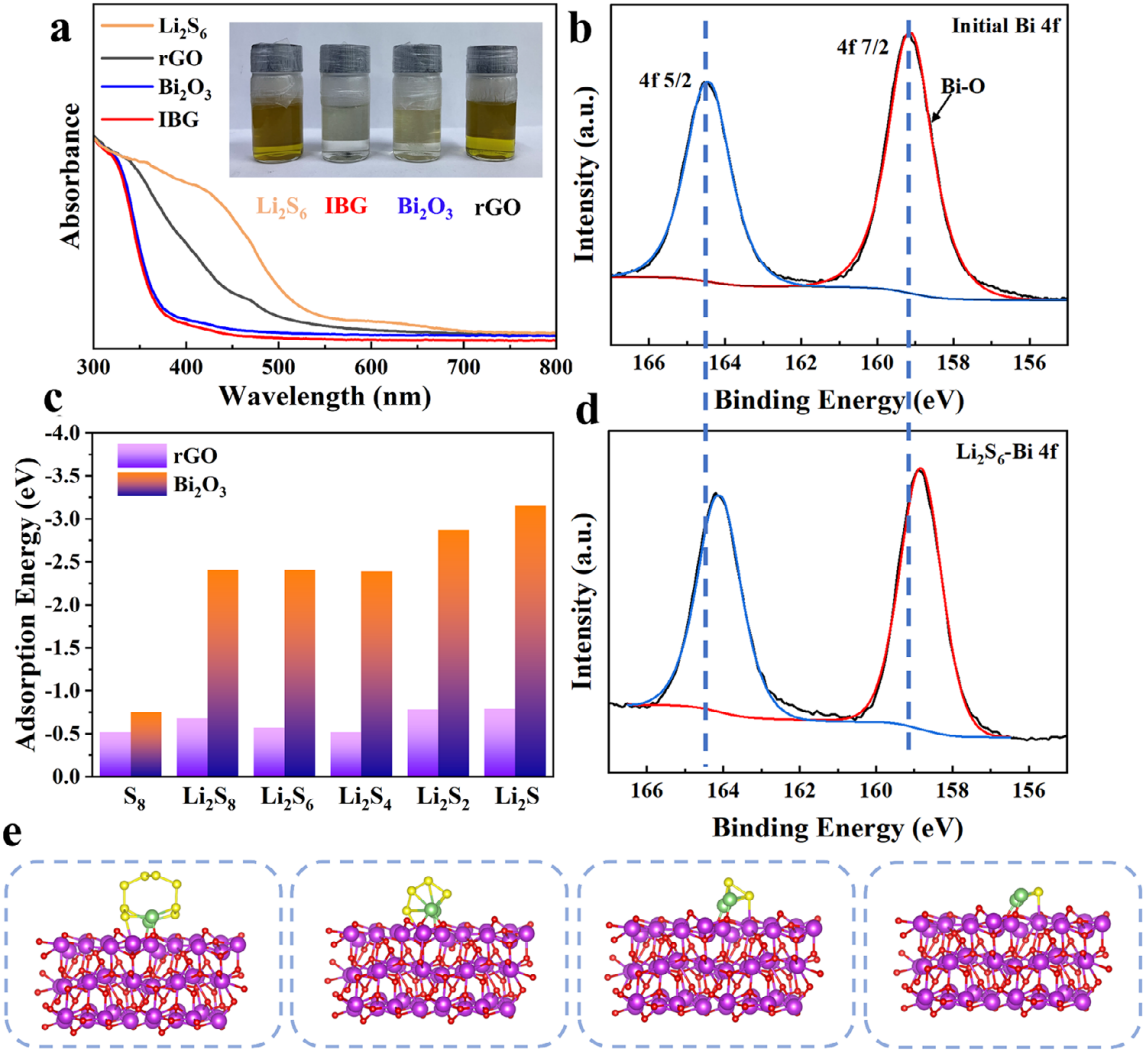
Adsorption of the soluble LiPSs. a) UV–vis spectra and inset: visual discrimination of Li_2_S_6_ solutions contained blank, IBG, Bi_2_O_3_, and rGO at room temperature; XPS spectra of Bi 4f of the IBG sample before b)/after d) adsorption of the LiPSs; c) adsorption energy of different LiPSs species (Li_2_S_8_, Li_2_S_4_, Li_2_S_2_, and Li_2_S) on the surface of rGO and IBG; e) optimized adsorption structures of different LiPSs species on the surface of IBG.

The “shuttle effect” of LiPSs is a key factor causing the rapid capacity decay in LSBs. This effect results from irreversible reactions that deplete the available active material with each cycle, leading to a gradual capacity fade. Therefore, the adsorption capacity of cathode electrodes for LiPSs is crucial for improving the performance and longevity of LSBs. Here, we investigated the interaction of IBG with LiPSs using UV–visible spectroscopy, XPS, and density functional theory (DFT) calculations. UV–visible spectra reveal the interactions between various materials (rGO, Bi₂O₃, and IBG) and LiPSs (Li₂S₆). As shown in **Figure** **2**a, the Li₂S₆ solution exhibits characteristic absorption peaks. In the presence of rGO, the peak intensity decreases only slightly, indicating that rGO has a relatively weak adsorption capability for LiPSs. In contrast, Bi₂O₃ and IBG composites exhibit significantly reduced absorbance, indicating strong adsorption of LiPSs. These results suggest that the primary absorption ability of IBG toward LiPSs is attributed on the embedded Bi₂O₃ nanoparticles. Moreover, the visual experiment shown in the inset of Figure 2a further confirms this finding. After 1 h of adsorption, the solutions containing IBG and Bi_2_O_3_ become nearly colorless, whereas the solution with rGO shows almost no color change compared to the original solution. Upon closer observation, the solution with IBG is noticeably lighter than that with Bi_2_O_3_, implying that IBG has higher adsorption efficiency than Bi_2_O_3_ alone. This enhanced adsorption capacity may result from the synergistic effect between Bi₂O₃ and rGO, where rGO prevents the aggregation of Bi₂O₃, thus exposing more active sites to capture LiPSs. XPS analysis further highlights the chemical interactions between IBG and LiPSs. In the Bi 4f spectrum, before LiPSs adsorption (Figure 2b), two characteristic peaks at 164.6 and 159.3 eV, corresponding to Bi‐O bonds, confirm the presence of Bi₂O₃. However, after Li₂S₆ adsorption (Figure 2d), the Bi 4f peaks shift significantly to lower binding energy, attributed to the interaction between the lone pair electrons of Bi ions and S. Since the electronegativity of Bi ions is higher than that of S, after interacting with LiPSs, the electron cloud density of Bi increases. Comparing the initial spectrum with the post‐adsorption spectrum further confirms that Bi₂O₃ has a strong affinity for LiPSs. DFT calculations further verify the adsorption capability of various sulfur species (S₈, Li₂S₈, Li₂S₆, Li₂S₄, Li₂S₂, and Li₂S) on the surfaces of rGO and Bi₂O₃ (Figure 2c; Table S1, Supporting Information). The optimized adsorption structures are shown in Figure 2e. The results show that Bi₂O₃ exhibits higher adsorption energy for all sulfur species than rGO, with the adsorption sites primarily located on Bi ions, as confirmed by the XPS analysis. Particularly in comparison to the adsorption energy of rGO for S₈, Bi₂O₃ begins to show a strong binding affinity for LiPSs starting from Li₂S₈. These species are key intermediates in the LiPSs “shuttle effect” and play an indelible role in suppressing it. This indicates that the introduction of Bi₂O₃ not only promotes the physical adsorption of LiPSs but also provides chemical sites that stabilize the LiPSs. Meanwhile, rGO acts as a conductive framework that stabilizes Bi₂O₃ particles, preventing its aggregation, thereby enhancing the overall LiPSs capture capability of the composite material.

**Figure 3:**
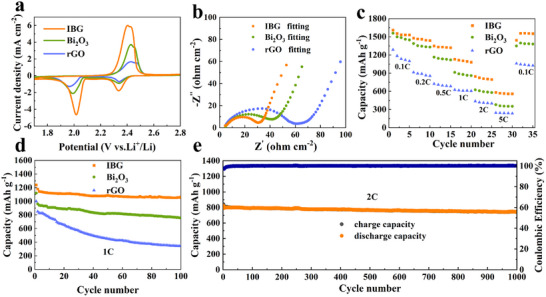
Electrochemical performance at room temperatures: a) cyclic voltammetry test of IBG, Bi_2_O_3,_ and rGO cells, b) Nyquist plots of above cells before cycling, c) rate capability of above cells, d) cycle performance at 1 C, e) long‐term cycle performance at 2 C.

To investigate the effect of the IBG cathode host on the electrochemical performance of LSBs at room temperature, S/IBG, S/Bi₂O₃, and S/rGO electrodes were synthesized using the traditional melt diffusion method. TGA presented in Figure S5 (Supporting Information) shows that the actual sulfur content in the three composites is ≈73.5%, 72.7%, and 74.4%, respectively. From the cyclic voltammetry (CV) curves (**Figure** **3**a), the IBG electrode material shows distinct redox peaks in the range of 1.7 to 2.8 V (vs Li⁺/Li). Compared to Bi₂O₃ and rGO alone, IBG shows sharper redox peaks, indicating that the IBG composite effectively improves the reaction kinetics of lithium ions. Especially at the reduction peak, the IBG electrode exhibits a stronger current response and a higher peak potential, indicating higher catalytic activity that promotes the conversion of LiPSs. From the electrochemical impedance spectra (EIS) shown in Figure 3b, one may see that the IBG electrode exhibits the lowest interfacial resistance, further confirming its promotion of lithium‐ion transport. Compared to Bi₂O₃ and rGO electrodes, the diameter of the semicircle for IBG is significantly smaller, suggesting a significant reduction in interfacial charge transfer resistance. This is attributed to the fact that Bi₂O₃ provides a larger active surface area that facilitates the charge transfer, while rGO offers a conductive network, further enhancing the overall electrochemical performance of the electrode. The rate performance test results (Figure 3c) show that IBG electrode exhibits excellent discharge capacity at various current densities. At 0.1C, it delivers a capacity of 1610 mAh g⁻¹, which is 96% of the theoretical value. At 2C, the initial discharge capacity is ≈800 mAh g⁻¹. Even at 5C, it still delivers ≈600 mAh g⁻¹ of reversible capacity, while rGO remains at only 245 mAh g⁻¹, which is lower than the theoretical capacity of the first discharge plateau (≈425 mAh g⁻¹). The cycling performance test results show that the IBG electrode exhibits excellent cycling stability at both 1C and 2C. Under 1C conditions (Figure 3d), its initial capacity is 1240 mAh g⁻¹, and after 100 cycles, the capacity remains at 1056 mAh g⁻¹, with a capacity retention rate of 85%. At 2C (Figure 3e), after 1000 cycles, its capacity remains above 746 mAh g⁻¹ with minimal capacity decay, showing excellent long‐cycle stability. At the same time, the Coulombic efficiency stays close to 100%, indicating good reversibility of the material.

**Figure 4:**
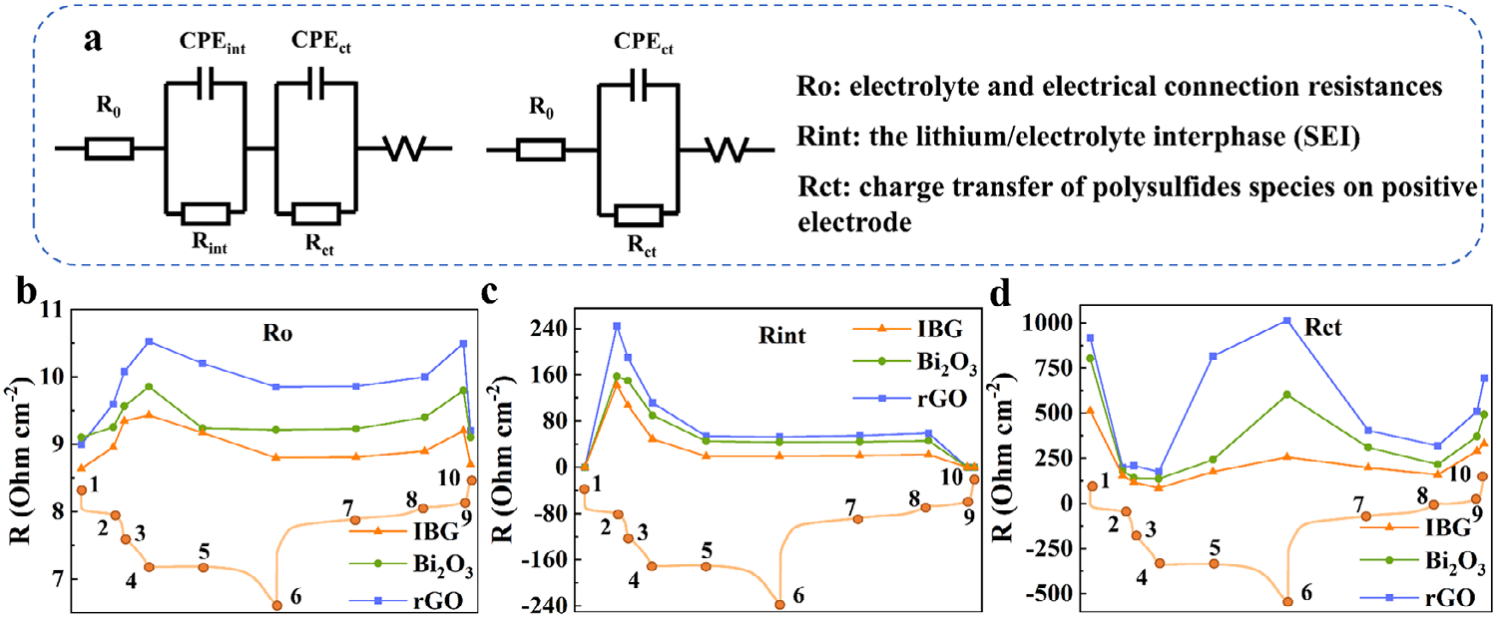
In situ EIS of the cells with IBG, Bi_2_O_3_, and rGO electrodes: a) the equivalent circuit of electrochemical impedance spectra. Evolution of the different resistance; b) *R_o_*, c) *R_int_*, and d) *R_ct_* at −20 °C values of cells with IBG, Bi_2_O_3_, and rGO.

Further, EIS was performed at low temperatures to characterize the electrochemical properties of the three cathodes at different stages of the charge‐discharge cycle. As shown in Figure S6 (Supporting Information), the numbers represent different stages in the charge‐discharge process. The equivalent circuit diagram is shown in **Figure** **4**a, where *R_o_* represents the electrolyte and electrical contact resistance, *R_int_* represents the interfacial resistance between lithium and electrolyte, and *R_ct_* represents the charge transfer resistance. Figure 4b shows the variation trends of *R_o_* for the three electrodes. For all three materials, *R_o_* fluctuates during the charge–discharge process, but it is evident that the IBG electrode shows a relatively low and stable *R_o_*, remaining between 8.5–9.5 Ω. The electrolyte resistance of the rGO electrode is as high as 9.0–11.0 Ω with larger fluctuations. This indicates that the IBG composite can provide more stable ion conduction pathways at low temperatures, reducing electrolyte resistance. This may be related to the conductive network provided by rGO and the enhanced interfacial charge transfer by Bi₂O₃. Figure 4c shows the variation trend of *R_int_*. During the first charge of the battery, a reaction occurs between the lithium anode and the electrolyte, resulting in the formation of an electrode/electrolyte interface (SEI) layers. This process usually increases interfacial resistance as the SEI film hinders lithium‐ion migration, thereby increasing the impedance between lithium and the electrolyte. As the battery continues to charge, the SEI film undergoes reorganization and repair, optimizing its structure while some unstable products are consumed. This enhances the electrochemical stability of the SEI films.^[^ ^40^ ^]^ At this point, the conductivity of the SEI films increases, leading to a reduction in resistance. As a result, *R_int_* initially increases and then decreases as charging and discharging proceed. The IBG electrode shows a significant decrease in *R_int_* during the charge‐discharge process, especially in the initial stage of discharge, where *R_int_* drops rapidly from ≈150 Ω to ≈20 Ω. This is closely related to the gradual reduction of sulfur. In contrast, the *R_int_* of rGO and Bi₂O₃ electrodes is relatively higher and decreases less significantly, remaining within the range of 60–70 Ω. This indicates that IBG materials can effectively optimize the structure of the SEI films and thus maintain low interfacial resistance, thereby facilitating faster lithium‐ion migration. Figure 4d shows the variation trend of *R_ct_* during charge–discharge cycles. *R_ct_* reflects the charge transfer resistance between the electrode and electrolyte, which is typically related to sulfur reduction and the formation of Li₂S. In our previous study, we found that, unlike at room temperature where the solid‐phase reaction stage is the rate‐determining step during discharge, at low temperatures, in addition to the increased *R_ct_* in the solid‐phase platform, the *R_ct_* of the Li₂S₈ to Li₂S₄ reaction in the liquid‐phase conversion process also increases sharply.^[^ ^35^ ^]^ This indicates that the Li₂S₈ to Li₂S₄ reaction is another process that affects the reaction kinetics at low temperatures. The *R_ct_* of IBG electrode decreases significantly during discharge. Notably, compared to the rGO one, the IBG electrode continues to show a decreasing trend in *R_ct_* on the second platform. However, the *R_ct_* of the rGO electrode rises to some extent (Figure S7, Supporting Information), indicating that IBG exhibits targeted catalytic activity in the rate‐determining step at low temperatures. At the end of discharge, the *R_ct_* of the IBG electrode remains low resistance at ≈250 Ω and stays between 200–300 Ω during the later stages of charging. The *R_ct_* of rGO is significantly higher, with a maximum reaching 800 Ω, possibly due to poor deposition of Li₂S. The Li₂S deposition layers on the IBG electrode may have good interfacial contact with the electrolyte. At the beginning of charging, solid Li₂S is gradually oxidized to lower‐order LiPSs, which increases the reactivity. At the same time, the solid Li₂S is gradually stripped, causing charge transfer resistance to decrease again. At this point, charge transfer becomes smoother. As charging continues, LiPSs are gradually reduced to elemental sulfur (S₈), and the concentration of higher‐order LiPSs decreases. Sulfur gradually deposits and the interfacial reaction becomes less active, causing impedance to increase. Furthermore, as sulfur precipitates out of the electrolyte, the transport pathways at the interface are blocked, further increasing the charge transfer resistance. However, due to the good deposition layers and low reaction energy barrier of the IBG electrode, it maintains a low impedance during this stage. Therefore, the *R_int_* and *R_ct_* of IBG at low temperatures are significantly lower than those of rGO and Bi₂O₃. This indicates that it has a significant promoting effect on the electrochemical reactions in LSBs. Especially during the charge‐discharge process, the IBG electrode exhibits lower charge transfer resistance. This supports its key role in targeted catalysis during the conversion of Li₂S₈ to Li₂S₄.

**Figure 5:**
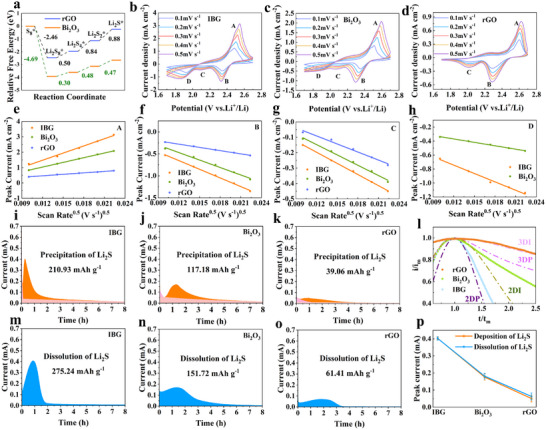
Lithium‐ion transfer kinetics and Li_2_S deposition: a) Gibbs free energy profiles of LiPSs on the surface of rGO and IBG; b–d) CV profiles of the LSBs with IBG, Bi_2_O_3,_ and rGO as sulfur host materials at different scan rates from 0.1 to 0.5 mV s^−1^; e‐h) the plots of peak intensity against the square root of scan rate (*I_p_‐ν^1/2^*) extracted from the peak (A, B, C, D) currents in Figure 5b,c,d. l) corresponding dimensionless transient in comparison with theoretical nucleation models. *i_m_*: peak current; *t_m_*: time needed to achieve the peak current; i–k) potentiostatic discharge curves of Li_2_S_8_ solution at 2.05 V on the IBG, Bi_2_O_3_ and rGO electrodes. m–o) The dissolution of Li_2_S capacity for IBG, Bi_2_O_3_ and rGO. p) The comparison of peak currents for the deposition and dissolution of Li_2_S on three different electrodes. (*n* = 3; **p ≤ 0.01, ***p ≤ 0.001 for variables of significance).

The free energy variation diagram in **Figure** **5**a shows the reaction energy barriers between S₈ and Li₂S, which are used to explore the catalytic ability of IBG for sulfur conversion reactions. It can be seen that IBG exhibits significant differences in reaction pathways and energy changes during sulfide conversion. The free energy curve of rGO is significantly higher, indicating that its reaction pathway has higher energy barriers and requires more energy to complete LiPSs conversion. In contrast, Bi₂O₃ shows lower reaction barriers, especially during the Li₂S₈ to Li₂S₄ and Li₂S₄ to Li₂S₂/Li₂S reactions. This indicates that IBG can significantly accelerate the rate‐determining steps of these two sulfur conversion reactions, providing catalytic support for the LiPSs reactions. To verify the catalytic effect of IBG on sulfur conversion reactions at low temperatures, CV tests were conducted on the three electrodes. Figures 5b–d show the current‐voltage relationships of IBG, Bi₂O₃, and rGO electrodes at different scan rates. It can be observed that the IBG electrode exhibits more prominent redox peaks under low‐temperature conditions, and the reduction peaks of the IBG electrode show higher and sharper peak potentials. This indicates that IBG has faster reaction kinetics and can more effectively promote the LiPSs conversion. In contrast, rGO shows more passivated peaks and the third reduction peak corresponding to the further reduction of Li₂S₂ and Li₂S no longer appears, indicating weaker catalytic ability at low temperatures. In the relationship graphs between scan rate and peak current (Figures 5e–h), the peak current of IBG shows a linear relationship with the square root of the scan rate. The lithium‐ion diffusion coefficient D_Li+_ (cm^2^ s⁻¹) is calculated from the slope of the fitted line and shown in Table S2 (Supporting Information). At different stages, the slope of the IBG electrode is larger than those of the other two control materials. This suggests that the IBG composite can significantly accelerate the diffusion speed of LiPSs under low‐temperature conditions. In contrast, the D_Li+_ of Bi₂O₃ electrode in the reduction peak corresponding to the rate‐determining step of Li₂S₈ to Li₂S₄ conversion is similar to that of IBG. However, rGO electrodes show significant differences, indicating that Bi₂O₃ nanoparticles promote the conversion of Li₂S₈ to Li₂S₄. But relying solely on Bi₂O₃ or the rGO network is insufficient to accelerate Li₂S conversion at low temperatures. Further potentiostatic nucleation experiments of Li₂S were conducted on IBG, Bi₂O₃, and rGO electrodes to test the catalytic ability of different host materials in the Li₂S₄ to Li₂S₂/Li₂S reaction process. Figure 5l shows a comparison between four classic electrochemical deposition models and i‐t images,^[^ ^41^, ^42^ ^]^ where the response time and current are converted to dimensionless forms. The four electrochemical deposition models are the Scharifker‐Hills models: 3D instantaneous (3DI) and 3D progressive (3DP) nucleation models, followed by the Bewick, Fleischman, and Thirsk models: 2D instantaneous (2DI) and 2D progressive (2DP) models (Table S3, Supporting Information). After comparison, it is found that Li₂S nucleation at low temperatures in IBG‐based batteries is more inclined toward the 2DP model. Bi₂O₃ and rGO tend toward the 3DP and 3DI models, meaning that the initial nucleation rate (instantaneous) in rGO‐based batteries is higher than in IBG‐ or Bi₂O₃‐based batteries (progressive). In other words, rGO‐based batteries tend to form more initial nuclei, which inevitably leads to disordered deposition. On IBG electrode, fewer initial nuclei are formed, corresponding to the 2DP model, resulting in larger Li₂S grains on the cathode and longer coverage times, which makes the Li₂S₄ to Li₂S₂/Li₂S reaction more complete. Figures 5i–k clearly show the deposition amounts of Li₂S on the IBG, Bi₂O₃, and rGO electrodes. The IBG electrode shows the highest Li₂S deposition amount, reaching 210.03 mAh g⁻¹, which is much higher than Bi₂O₃’s 117.18 mAh g⁻¹ and rGO's 39.06 mAh g⁻¹. Furthermore, the solid‐liquid conversion of Li_2_S to Li_2_S_4_ was also examined using the potentiostatic charge process after galvanostatic discharge (Figures 5m–o). The charge and discharge process of LSBs typically involves lithium ions reacting with sulfur to form Li₂S, followed by a reverse process in which Li₂S is converted back to sulfur. At lower temperatures, the reaction rate between lithium ions and Li₂S in the electrolyte slows down, leading to a slower dissolution process of Li₂S. As a result, at low temperatures, the insufficient re‐dissolution of Li₂S prevents its rapid recovery into usable polysulfides, leading to incomplete restoration of active materials during the charging process and thus affecting the overall performance and cycle life of the battery. IBG displays the increased dissolution capacity (275.24 mAh g^−1^) compared with those of Bi_2_O_3_ (151.72 mAh g^−1^) and rGO (61.41 mAh g^−1^), which reflects that IBG also significantly promotes the kinetics of Li_2_S dissolution. Figure 5p illustrates a comparison of the peak currents for the deposition and dissolution of Li₂S on three different electrodes. During the deposition and dissolution processes of Li₂S, the IBG (0.40/0.40 mA) electrode exhibits the highest peak currents compared to BI₂O₃ (0.17/0.16 mA) and RGO (0.05/0.07 mA), indicating that IBG significantly enhances the deposition and dissolution kinetics of Li₂S. Those result strongly indicates that the island‐like structure of IBG can significantly enhance the Li₂S deposition capacity at −20 °C, effectively suppressing LiPSs diffusion loss. This remarkable catalytic performance not only helps to improve the capacity of LSBs, but also, due to the increased exposure area of the deposition layer, promotes the dissolution efficiency of Li₂S, effectively extending the battery's cycle life.

**Figure 6:**
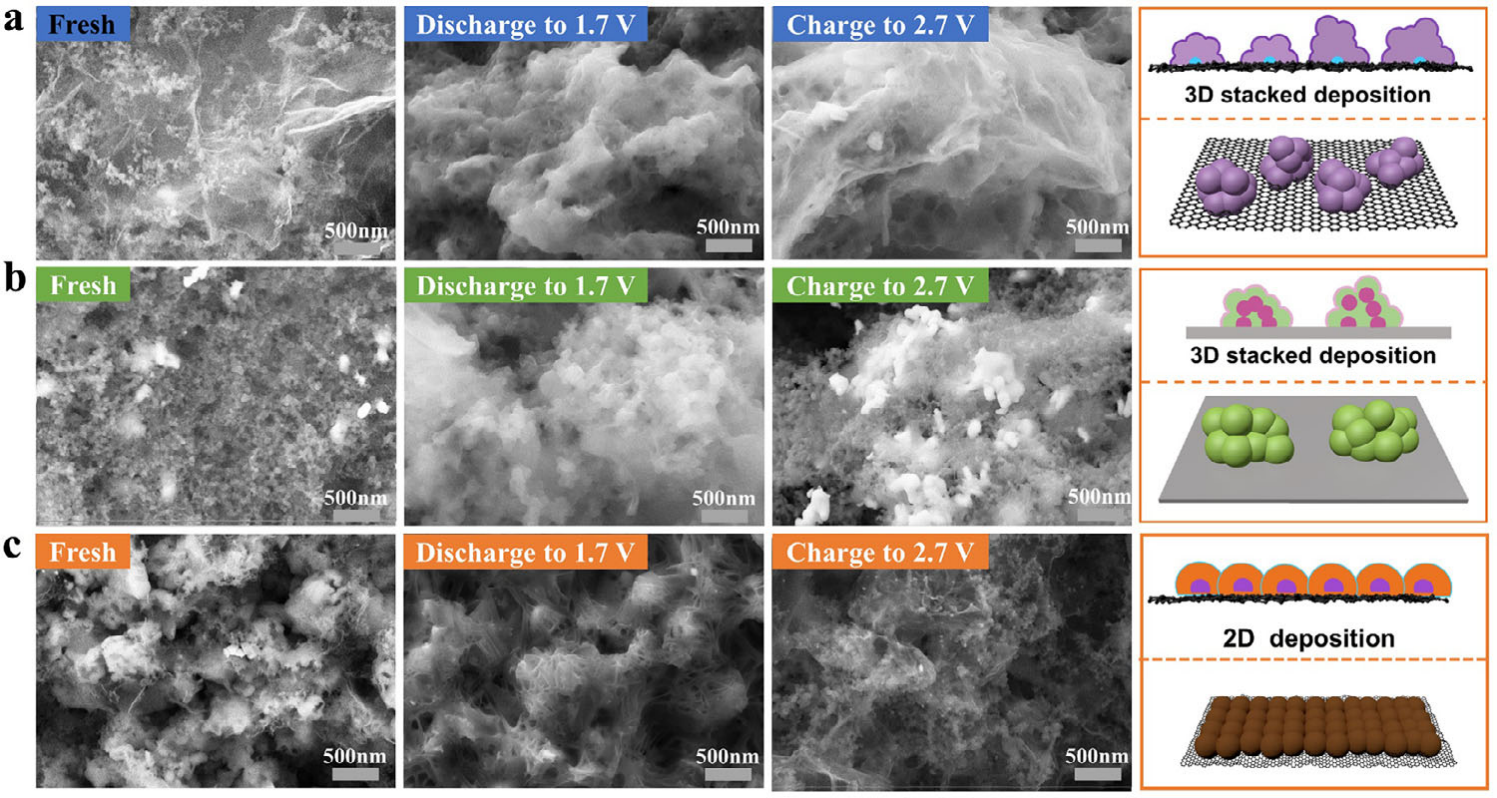
Deposition behavior of Li_2_S. SEM images of various electrodes at different charging and discharging stages: a) rGO, b) Bi_2_O_3_, and c) IBG.

To visualize the specific details of Li₂S deposition, SEM scans were performed on the three cathodes before charge–discharge, and after discharge at 1.7 V and charge at 2.7 V. **Figures** **6**a–c correspond to the Li₂S deposition morphology of rGO, Bi₂O₃, and IBG electrodes at different stages, respectively, with a schematic of the morphology shown on the right. In the initial state, the rGO surface shows a loose structure with high porosity, but the surface is not uniform, with some localized clusters. This uneven morphology may provide initial nucleation sites for subsequent Li₂S deposition, but due to the lack of effective deposition control, Li₂S tends to form disordered 3D stacking at these sites. The Bi₂O₃ surface is more compact and uniform but with fewer pores. Although this compact structure has lower initial porosity than rGO, it provides a more stable surface, conducive to uniform LiPSs deposition. IBG exhibits a distinct island‐like structure, with a more dispersed and uniform surface, showing good porosity. This island structure combines the high porosity of rGO with the catalytic activity of Bi₂O₃, providing more nucleation sites and helping to control the Li₂S deposition, preventing excessive 3D stacking. After discharge at 1.7 V, a large‐scale 3D stacking structure is clearly observed on the rGO surface. This 3D stacking of Li₂S leads to rapid aggregation of Li₂S particles, forming uneven large particles. This uncontrolled deposition reduces the effective surface area of the electrode, hinders ion and electron transfer, and leads to a decline in the electrochemical performance of LSBs in subsequent cycles. On Bi₂O₃ electrode, Li₂S deposition mainly follows the 3D progressive (3DP) nucleation model. This indicates that the initial nucleation rate on the Bi₂O₃ electrode is relatively slow, and the Li₂S deposition process proceeds gradually. Compared to rGO, fewer Li₂S particles are formed in Bi₂O₃, but the particles gradually accumulate, showing some 3D stacking characteristics. SEM images show that Li₂S particles on the Bi₂O₃ surface are relatively uniform, even some stacking occurs, indicating that despite Bi₂O₃’s catalytic properties, its control over Li₂S deposition is somewhat limited. The deposition of Li₂S still mainly takes a 3D form, which could cause some Li₂S particles to resist complete oxidation in subsequent charging stages. IBG's Li₂S deposition tends to follow the 2DP nucleation model. In this model, the initial nucleation of Li₂S is slower, and the particles gradually grow to form larger grains. This process ensures that Li₂S deposition is more uniform and smoother, and less likely to form disordered 3D structures. SEM images show that the particles on the IBG surface are more dispersed and do not form obvious 3D stacking structures, confirming its ability to effectively suppress excessive Li₂S accumulation. This smooth deposition facilitates electron and lithium‐ion transport, thereby improving the discharge efficiency and reaction rate of LSBs. After a charge at 2.7 V, the rGO electrode surface still retains a significant Li₂S deposition layer, indicating low oxidation efficiency of rGO material, which cannot fully oxidize Li₂S to S₈. This residual Li₂S layer not only affects the battery's capacity recovery but also increases electrode surface polarization with cycling, reducing the battery's cycling stability. In contrast, the Li₂S deposits on the Bi₂O₃ surface are more thoroughly oxidized compared to rGO. The images show less residual Li₂S, indicating that Bi₂O₃ has better oxidation catalytic ability, promoting Li₂S redox reactions and improving electrode reversibility. However, due to some stacking during Li₂S deposition, residual particles may remain after multiple charge‐discharge cycles. The Li₂S on the IBG electrode surface is almost completely oxidized, showing a clean morphology. This is significantly different from the rGO and Bi₂O₃ electrodes, indicating that IBG material has higher catalytic efficiency. The catalytic role of Bi₂O₃ accelerates the oxidation of Li₂S to S₈, while the conductive network of rGO provides pathways for electron transport. This near‐complete oxidation ensures high electrode reversibility during charge–discharge cycles, significantly improving the cycling performance and capacity retention of the battery. Through morphology analysis of the three electrodes in three states, it can be concluded that the Li₂S deposition mode of rGO and Bi₂O₃ electrodes is mainly 3D stacking. Particularly, rGO tends to form large deposits during discharge, affecting electrode activity. IBG's unique island‐like structure not only controls Li₂S deposition during discharge but also enables efficient oxidation reactions during charging. This structure takes advantage of both the conductivity of rGO and the catalytic activity of Bi₂O₃, forming a composite material with high conductivity and catalytic activity, significantly enhancing the electrochemical performance of LSBs at low temperatures.

**Figure 7:**
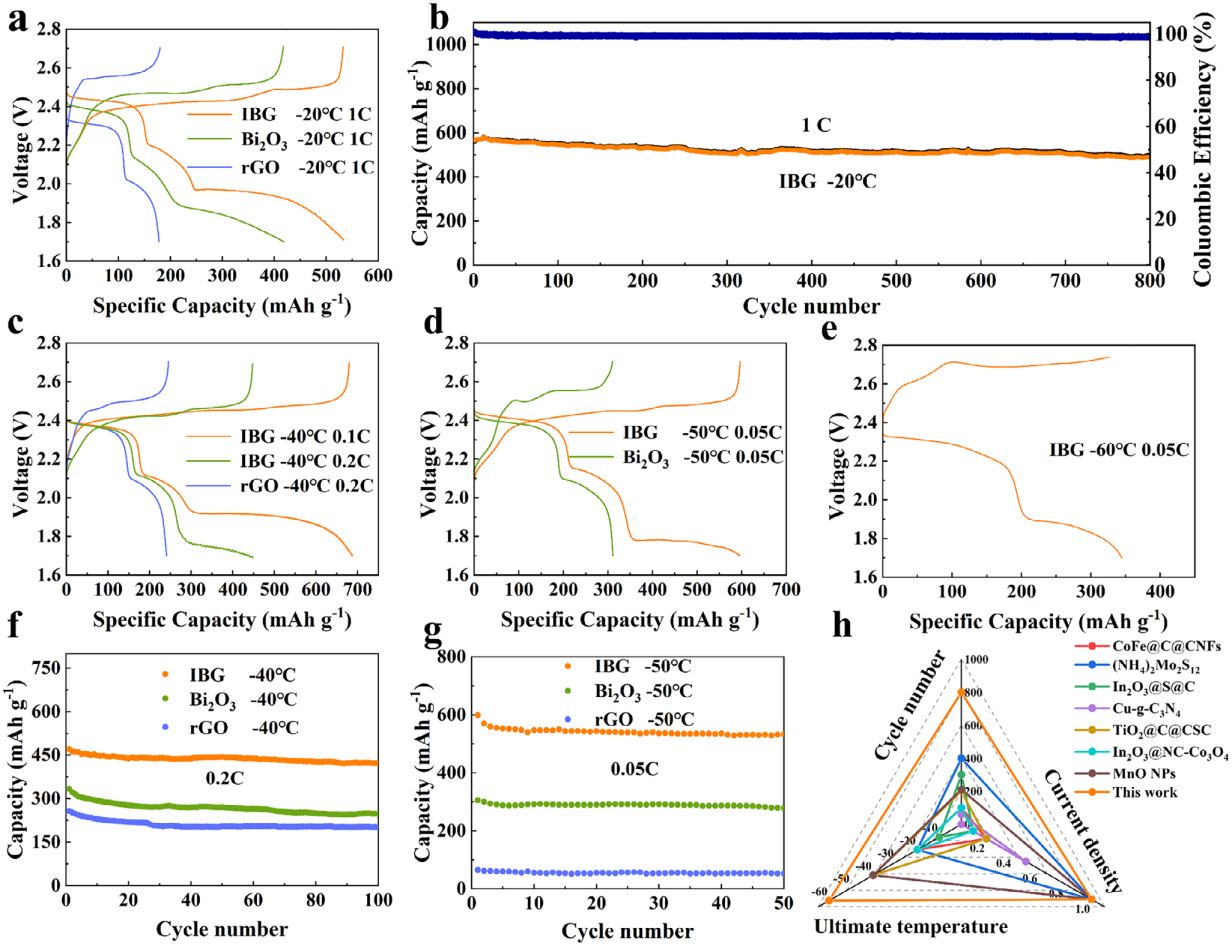
Electrochemical performance at low temperatures: a) first charge and discharge voltage profiles of IBG, Bi_2_O_3_, and rGO cells at 1 C and −20 °C; b) long‐term cycle performance of the cell with IBG at −20 °C; c) charge and discharge voltage profiles of IBG and rGO cells at 0.1 and 0.2 C at −40 °C; d) cycle performance of IBG and rGO cells of 0.2C at −40 °C; e) charge and discharge voltage profiles of IBG and Bi_2_O_3_ cells of 0.05 C at −50 °C; f) cycle performance of IBG and Bi_2_O_3_ cells of 0.05 C at −50 °C; g) first charge and discharge voltage profiles of IBG, cells of 0.05 C at −60 °C; h) radar map shows a comparison of properties between IBG and other reported cathode materials.

Based on the previous characterization of IBG electrode catalytic performance, IBG cathode modification materials are expected to have excellent application prospects in low‐temperature LSBs. At −20 and 0.1 °C rate, the discharge capacity of the IBG electrode is 887 mAh g⁻¹, significantly greater than those of Bi₂O₃ (718 mAh g⁻¹) and rGO (602 mAh g⁻¹) electrodes (Figure S8, Supporting Information), in addition, the IBG electrode also shows the smallest battery polarization and a flatter plateau. When the current density increases to 1 C, IBG and Bi₂O₃ electrodes exhibit high discharge specific capacities (**Figure** **7**a) of 548 and 425 mAh g⁻¹, respectively, but the IBG electrode maintains a flatter voltage plateau. The rGO electrode, in contrast, can hardly undergo an effective solid‐phase reaction under these conditions, with an initial capacity of less than 200 mAh g⁻¹ at °20 °C. This indicates that the IBG electrode can effectively catalyze the conversion of Li₂S₄ to Li₂S₂/Li₂S under low‐temperature conditions, reducing electrochemical polarization and maintaining good reaction reversibility and ion transport capability. rGO itself cannot effectively promote sulfur conversion reactions, especially at low temperatures, where its catalytic ability is limited. After 800 cycles at this current density, the IBG electrode exhibits a very high‐capacity retention rate, with an initial capacity of ≈570 mAh g⁻¹, and after 800 cycles, the capacity remains above 480 mAh g⁻¹ (Figure 7b), with a retention rate of ≈87%. The Coulombic efficiency consistently stays close to 100%, indicating good reversibility and excellent cycling stability at low temperatures. This performance can be attributed to the IBG providing good charge transfer pathways under low temperatures and catalyzing the two rate‐determining steps in low‐temperature LSBs, further enhancing sulfur conversion efficiency. When the temperature drops to °40 °C (Figure 7c), the specific capacities of the IBG electrode are 690 and 450 mAh g⁻¹ at 0.1C and 0.2C, respectively. The discharge‐specific capacity of the Bi₂O₃ electrode is only ≈560 mAh g⁻¹ at −40 and 0.1 °C (Figure S9, Supporting Information), significantly lower than that of IBG. This indicates that IBG can maintain the LiPSs conversion reaction in extremely low‐temperature environments, promoting the stepwise reduction of Li₂S₈ to Li₂S₄. Although Bi₂O₃ has some catalytic ability, due to limitations in its conductive network and severe deposition accumulation, it is insufficient to support complete liquid‐solid conversion. The performance of rGO at −40 °C is extremely poor, with a discharge specific capacity of less than 250 mAh g⁻¹ at 0.1C current density, indicating that rGO's conductivity is severely limited at low temperatures, and due to limited catalytic ability, it cannot effectively support sulfur conversion. After 100 cycles at 0.2C (Figure 7d), the capacities of the three cells are 420, 280, and 210 mAh g⁻¹, respectively. Even as the temperature further drops to −50 °C (Figure 7e), the IBG electrode still exhibits a discharge capacity close to 600 mAh g⁻¹ at 0.05C. At this temperature, Bi₂O₃ electrodes cannot undergo the liquid‐solid conversion reaction of Li₂S₄ to Li₂S₂/Li₂S. This indicates that the IBG electrode can still promote LiPSs conversion at extremely low temperatures and ensure lithium ions conduction through the rGO conductive network. Even at this temperature, the rGO electrode is unable to complete the rate‐determining step in the liquid phase (Li₂S₈ to Li₂S₄) (Figure S10, Supporting Information). After 50 cycles at 0.05C (Figure 7f), the capacities of the three batteries are 530, 276, and 52 mAh g⁻¹, respectively. Finally, at −60 °C, IBG still demonstrates relatively high electrochemical activity (Figure 7g). Although the specific capacity decreases, it still reaches ≈350 mAh g⁻¹. Compared to other advanced LSBs, which generally cannot function at this temperature (Figure 7h; Table S4, Supporting Information), this is the current limit for LSBs. This further confirms that IBG can effectively lower the reaction energy barriers of the Li₂S₈ to Li₂S₄ rate‐determining step and maintain conductivity through the rGO network. This enables IBG to still perform well in lithium ions conduction and LiPSs conversion under low‐temperature conditions.

## 3 Conclusion

This study designed a nanospherical Bi₂O₃ composite material embedded in rGO as the sulfur host for LSBs and systematically evaluated its electrochemical performance at different temperatures. First, Li₂S₆ adsorption experiments and UV–vis spectroscopy tests demonstrated IBG's strong adsorption ability to soluble LiPSs, effectively suppressing the “shuttle effect” and therefore reducing the likelihood of side reactions between LiPSs and the lithium anode. Meanwhile, theoretical calculations showed that this composite can significantly reduce the energy barriers for LiPSs conversion, promoting LiPSs conversion reactions. CV and EIS tests further confirmed that IBG material has low charge transfer resistance and excellent reaction kinetics, effectively promoting the reduction of Li₂S₈ to Li₂S₄. Compared to Bi₂O₃ and rGO materials, IBG, due to its unique island structure, could effectively suppress the Li₂S accumulation, increase deposition capacity (Li₂S₄ to Li₂S reduction), and form a large electrode‐electrolyte interface, further promoted Li₂S dissolution and oxidation. As a result, LSBs maintained excellent sulfur utilization and cycling stability under high current densities and low temperatures. At −220 °C and 0.1C current density, IBG delivered a specific capacity close to 900 mAh g⁻¹, with a Coulombic efficiency near 100%. At extremely low temperatures of −40 and −50 °C, IBG not only maintained its high efficiency in LiPSs adsorption and catalytic conversion but also played a key catalytic role in the conversion of Li₂S₈ to Li₂S₄. Remarkably, at −60 °C, IBG‐based LSBs can still operate normally with a rather high capacity value. In summary, as a sulfur host material for LSBs, IBG, with its excellent LiPSs adsorption ability, low conversion energy barriers, and effective control over Li₂S deposition, exhibited outstanding electrochemical performance at room temperature and maintains stable cycling behavior under low‐temperature conditions due to its catalytic effect on two rate‐determining steps. These findings provide new ideas for developing efficient low‐temperature LSBs materials and demonstrate IBG's broad application prospects in extreme environments.

## Conflict of Interest

The authors declare no conflict of interest.

## Supporting information



Supporting Information

## Data Availability

The data that support the findings of this study are available from the corresponding author upon reasonable request.
