# Supplementary material for: Overcoming the Low‐Temperature Barrier: Controlling Li₂S Deposition and Enhancing Catalysis in Lithium‐Sulfur Batteries Using Island‐like Bi₂O₃ on rGO
Source: Adv Sci (Weinh). 2025 Mar 17;12(18):2502045. doi: 10.1002/advs.202502045 (PMC12079457; doi:10.1002/advs.202502045)
Supplement: Supplementary file 1 — Supporting Information [file ADVS-12-2502045-s001.docx]

Supporting Information

**Overcoming the Low-Temperature Barrier: Controlling Li₂S Deposition and Enhancing Catalysis in Lithium-Sulfur Batteries Using Island-like Bi₂O₃ on rGO**

*Hai-Ji Xiong, Ding-Rong Deng*, Yu-Lin Luo, Jia-Xi Song, Jin-Wei Yan, Shuang-Lin Cai, Jia Liang, Cheng-Wei Zhu, Ye Zeng, Gui-Fang Li, Yi Li, Wen-Jun Zhang*, Mei-Lin Liu* and Qi-Hui Wu**

H. Xiong, D. Deng, Y. Luo, J. Song, J. Yan, S. Cai, J. Liang, C. Zhu, Q. Wu,

College of Marine Equipment and Mechanical Engineering Key Laboratory of Energy Cleaning Utilization Development Cleaning Combustion and Energy Utilization Research Center of Fujian Province Xiamen Key Laboratory of Marine Corrosion and Smart Protective

Materials, Jimei University, Xiamen, Fujian 361021, China

E-mail: [drdeng@jmu.edu.cn](mailto:drdeng@jmu.edu.cn); [qihui.wu@jmu.edu.cn](mailto:qihui.wu@jmu.edu.cn)

Y. Li
Jiangsu Key Lab of Advanced Functional Polymer Design and Application, Department of Polymer Science and Engineering, College of Chemistry, Chemical Engineering and Materials Science, Soochow University, Suzhou 215123, China

W. Zhang
City Univ Hong Kong, Dept Mat Sci & Engn, Ctr Super diamond & Adv Films COSDAF, 83 Tat Chee Ave, Hong Kong 999077, Peoples R China

E-mail: [apwjzh@cityu.edu.cn](mailto:apwjzh@cityu.edu.cn)

M. Liu
School of Materials Science & Engineering, Center for Innovative Fuel Cell and Battery Technologies, Georgia Institute of Technology, Georgia 30332-0245, USA

E-mail: [meilin.liu@mse.gatech.edu](mailto:meilin.liu@mse.gatech.edu)

**Experimental Section**

**Synthesis**

*Synthesis of IBG.* All chemicals were analytical grade and used without further purification. 1 mmol of bismuth nitrate pentahydrate and 250 mg of PVP10000 were dispersed in 5 ml of 1 mol L^-1^ nitric acid solution, and then stirred for 0.5 hours in a Magnetic stirrer to obtain a uniform solution A. 50 mg of reduced graphene oxide was dispersed in 30 ml of organic solvent ethylene glycol, and sonicated for 1 hour to obtain a uniform solution B. Solution A was slowly added dropwise into solution B with stirring to obtain a uniform solution C. It was then placed in a 50 ml hydrothermal reactor and subjected to hydrothermal reaction at 150°C for 6 hours. After the reaction, the product was washed three times with ethanol and distilled water, respectively, then dried at 60°C in a drying oven. The dried product was thus calcined in a tube furnace at 500°C for 3 hours under a nitrogen atmosphere with a heating rate of 1°C min^-1^. The black powder obtained after calcination was IBG.

*Synthesis of Bi_2_O_3_.* The method is the same as the preparation of IBG, except that reduced graphene oxide was not added.

*Preparation of the Sulphur composites.* The S/IBG composite was prepared via the classical melt-diffusion method. First, the required amount of elemental sulfur and IBG (3:1 by mass) were mixed thoroughly by grinding. Then the mixture was heated at 155°C in a drying oven for 6 hours and then cooled naturally to room temperature. The S/Bi_2_O_3_, S/rGO composite was prepared by the same method as that of S/IBG.

*Polysulfide adsorption sample preparation.* Mix Li₂S and S (molar ratio 1:5) in 1,3-dioxolane/ethylene glycol dimethyl ether (DOL/DME, volume ratio 1:1) and heat at 60°C while stirring for 48 hours to obtain a Li₂S₆ solution (5×10^-4^ M). Then, 20 mg masses of IBG, Bi_2_O_3_, rGO are added to the 6 ml Li₂S₆ solution separately. Observe the color change of the solution and test the ultra-violet-visible absorption spectrum of the supernatant. The remaining solids are dried overnight for X-ray photoelectron spectroscopy (XPS) testing.

**Characterization of the materials**

X-ray diffraction (XRD) patterns of the samples were recorded on a Philips analytical X-pert diffractometer with Cu Ka radiation (λ = 0.1548 nm) at 40 kV and 30 mA and a step of 0.02. Data were recorded ranging from 10 to 80 degree. The scanning rate is 10℃ min^-1^. Scanning electron microscopy (SEM) was performed on a Zeiss Crossbeam 550 scanning electron microscope at an accelerating voltage of 15 kV. Transmission electron microscopy (TEM) observations were carried out FEI Tecnai F30 microscopes at 300 kV. Thermogravimetric analysis (TGA) was carried out on a Perkin Elmer instrument. Nitrogen adsorption and desorption isotherms at 77 K were characterized by a Micromeritics Tristar 3020 analyzer surface area and pore-size analyzer. Thermo Kalpha model X-ray photoelectron spectrometer was used to analyze the chemical interactions between the materials and polysulfides.

**Electrochemical measurements**

Dissolve 70 wt% active composite material, 20 wt% conductive agent (Super P), and 10 wt% binder (LA 5%) in a water-alcohol mixture. After ball-milling for 12 hours, the slurry is coated onto aluminum foil to prepare the cathode. The area of the aluminum foil is 13 mm and the sulfur loading is 1.2-1.5 mg cm^-2^. The electrolyte consists of 0.5 M LiCF₃SO₃ and 0.5 M LiNO₃ (dissolved in 1,2-dimethoxyethane and dioxolane in a 1:1 volume ratio, 35 μL). Battery cycling tests were conducted using a NEWARE BTS-5 V/20 mA battery tester (Shenzhen, China) with a voltage window of 1.7-2.8 V at room temper-ature. Cycling performance was tested at different rates (1 C = 1675 mA g⁻¹). The specific capacity was calculated based on sulfur as the active material. Cyclic voltammetry meas-urements and EIS data collection were carried out on a DH7000C workstation, with a frequency range of 0.1 Hz to 10 kHz.

**Calculation Details**

All the DFT calculations were conducted based on the Vienna Ab-inito Simulation Package (VASP). The exchange-correlation effects were described by the Perdew-Burke-Ernzerhof (PBE) functional within the generalized gradient approximation (GGA) method. The core-valence interactions were accounted by the projected augmented wave (PAW) method. The energy cutoff for plane wave expansions was set to 420 eV, and the 3×3×1 Monkhorst-Pack grid k-points were selected to sample the Brillouin zone integration. The vacuum space is adopted 15 Å above the surfaces to avoid periodic interactions. The structural optimization was completed for energy and force convergence set at 1.0×10^-5^ eV and 0.02 eV Å^-1^, respectively. Grimme’s DFT-D3 methodology was used to describe the dispersion interactions.

The adsorption energy (E_ads_) of adsorbate A was defined as

E_ads_ = E_(Li2Sx/substrate)_ - E_(Li2Sx)_ - E_(substrate)_

where E_(Li2Sx/substrate)_ represent the energy of Li_2_S_x_ (x = 1, 2, 4, 6, and 8) adsorbed on the surface. E_(substrate)_ is the energy of clean surface, E_(Li2Sx)_ represent the energies of Na_2_S_x_.

The Gibbs free energy changes (Δ*G*) of the reaction are calculated using the following formula:

∆G = ∆E + ∆ZPE - T∆S

where ΔE is the electronic energy difference directly obtained from DFT calculations, ΔZPE is the zero-point energy difference, T is the temperature and ΔS is the entropy change.

**Statistical analysis**

The data of peak currents for the deposition and dissolution of Li_2_S on three different electrodes is analyzed by two-sample t test. All statistical indications in the graph were presented as mean with standard deviation. Sample size (n) = 3 for experimental data. Statistical analysis results such as p value are described in each figure caption. A p value <0.05 was considered statistically significant. All statistical analyses were conducted using Origin 2022.


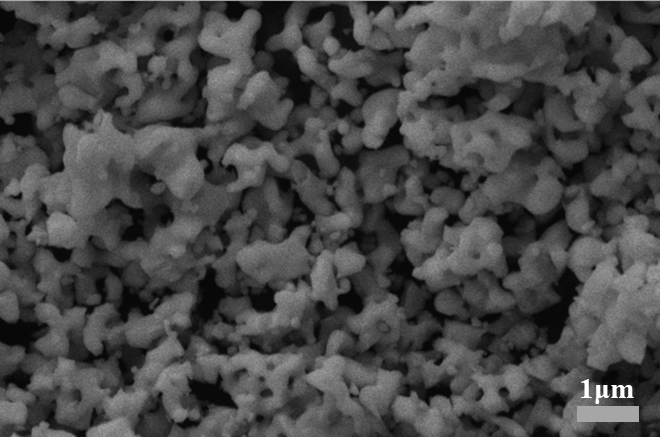


**Figure S1.** SEM images of the Bi_2_O_3_ sample


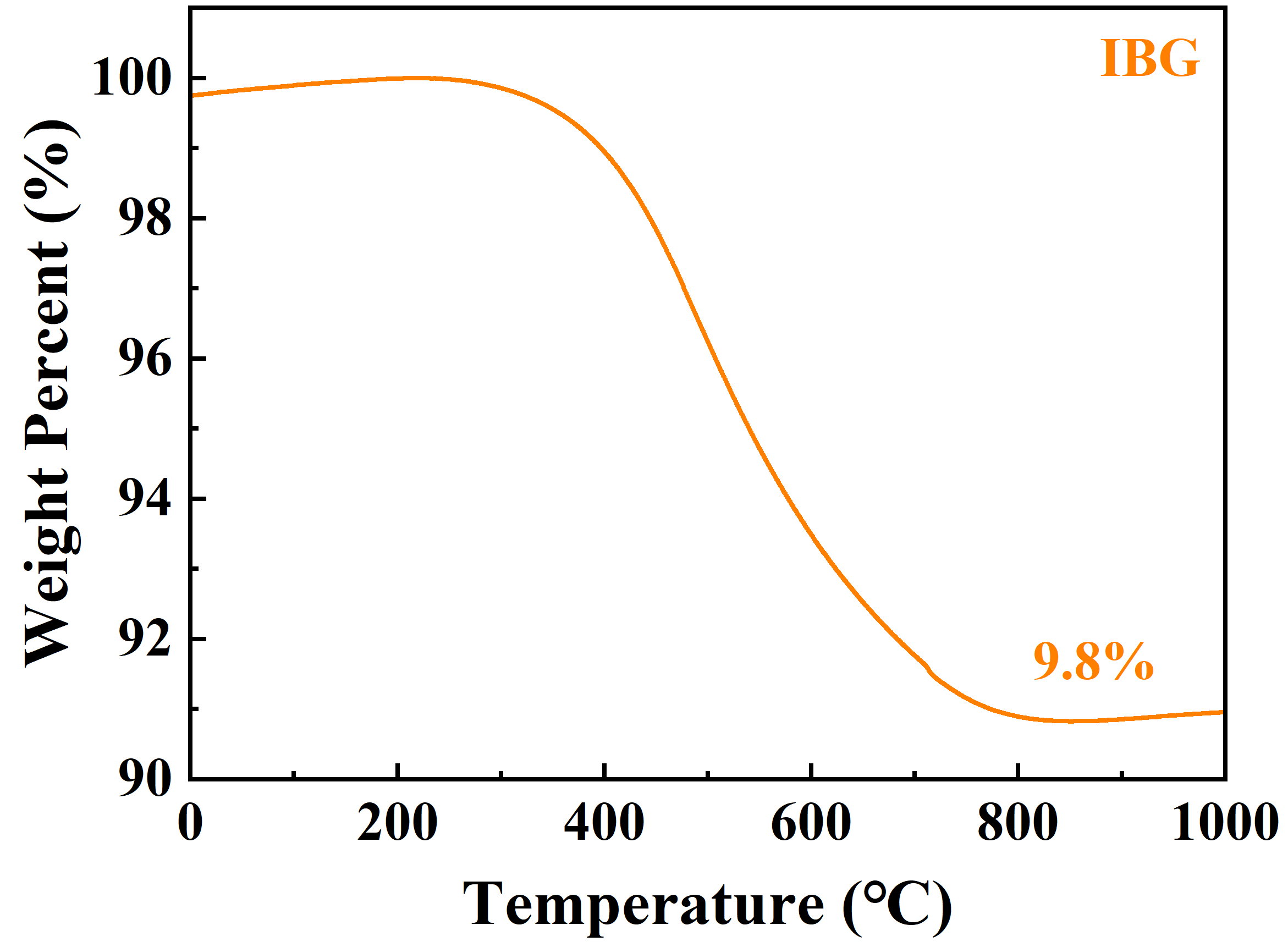


**Figure S2.** TGA of the IBG sample.


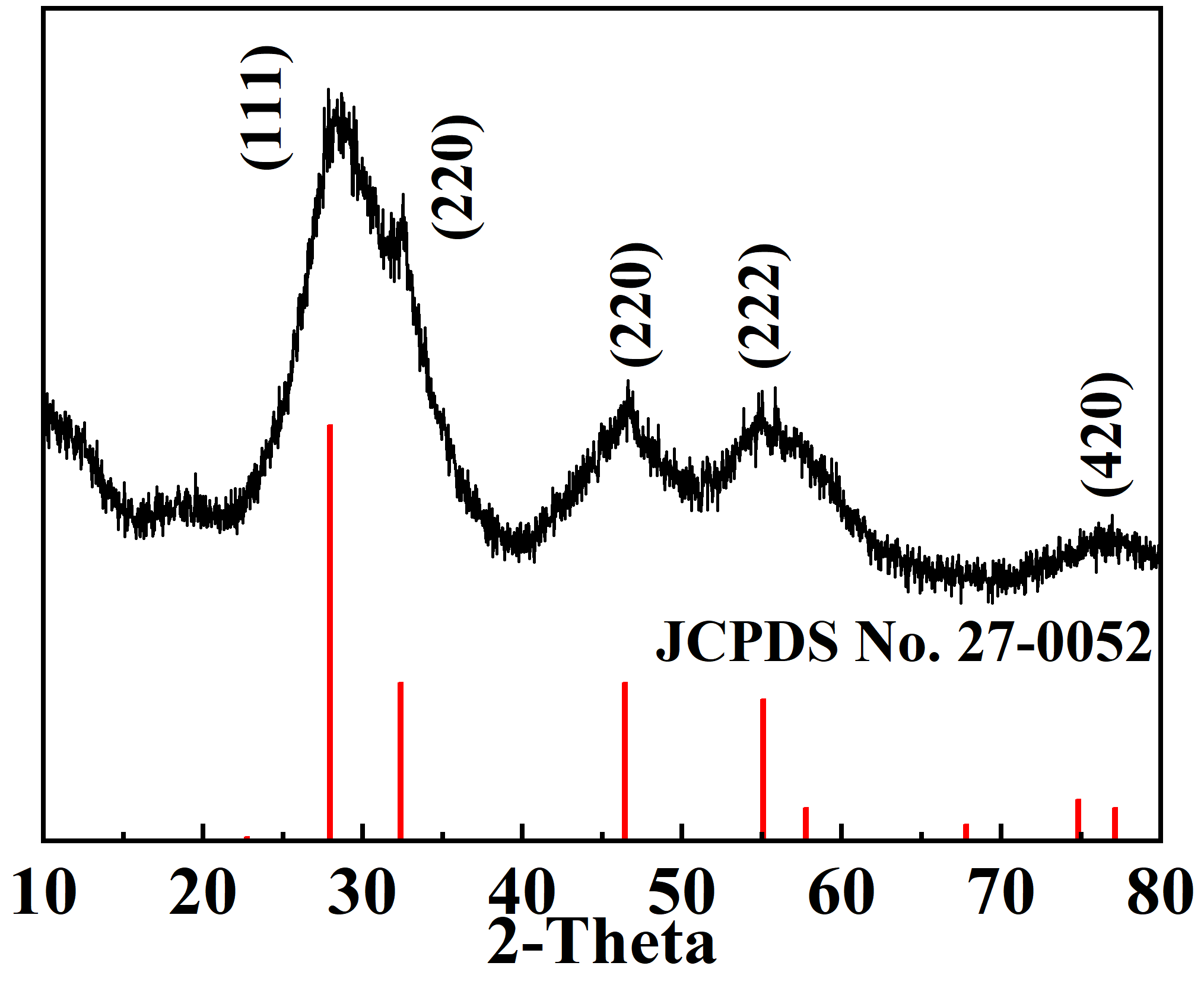


**Figure S3.** XRD patterns of the Bi_2_O_3_ sample.


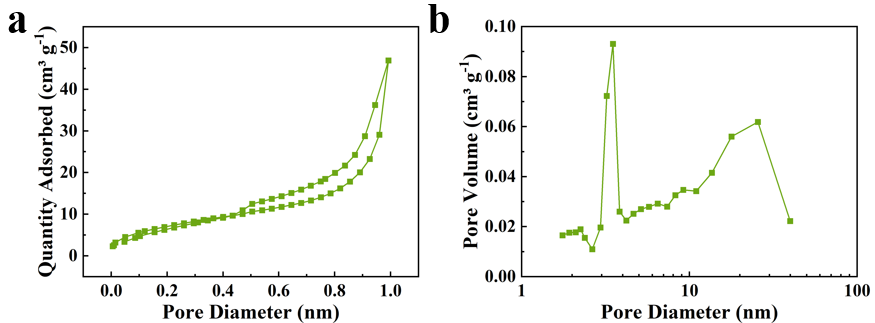


**Figure S4.** (a) nitrogen adsorption-desorption isotherms and (b) pore size distribution of the Bi_2_O_3_ sample.


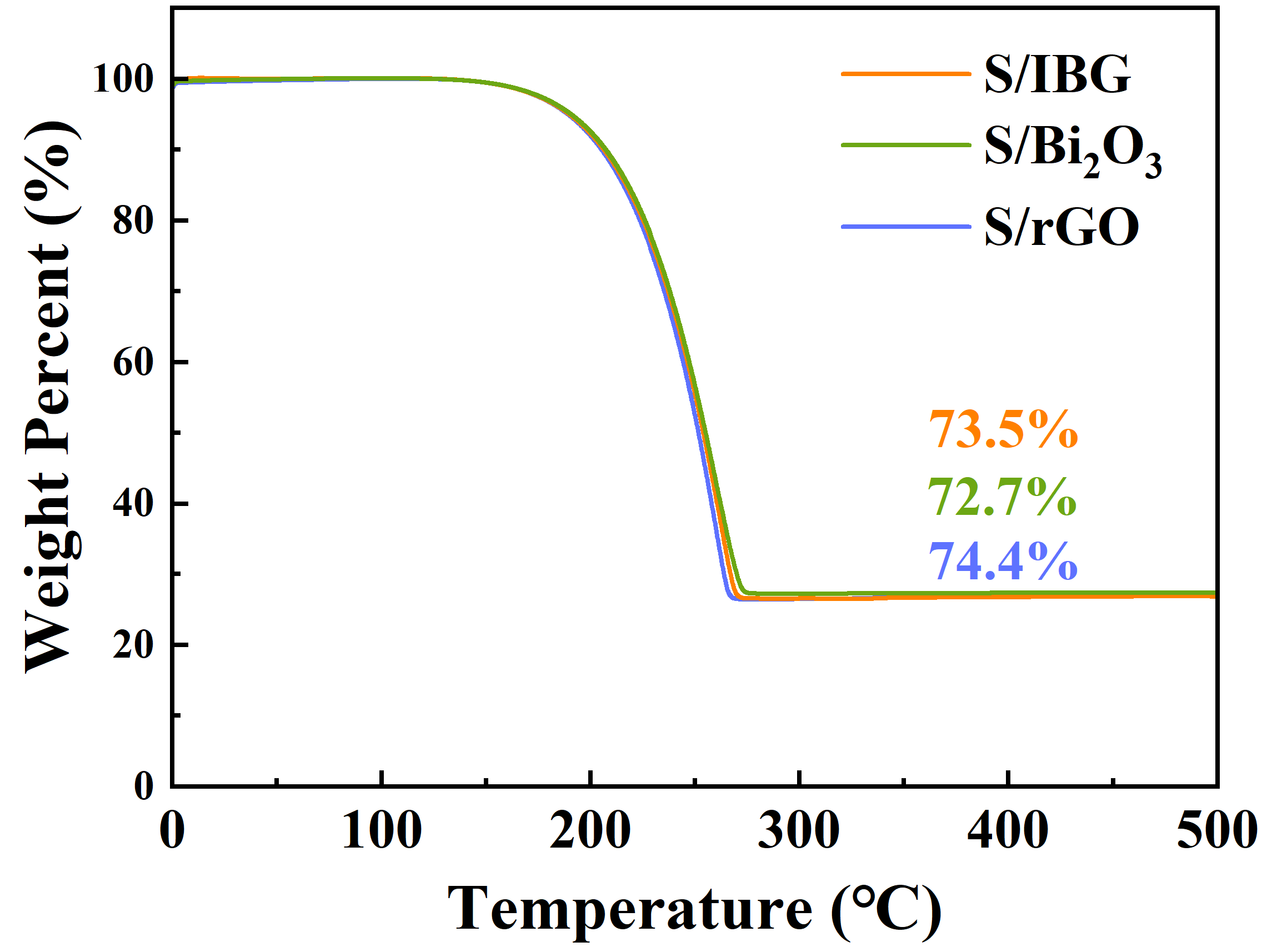


**Figure S5.** TGA of the S/IBG, S/Bi_2_O_3_ and S/rGO.


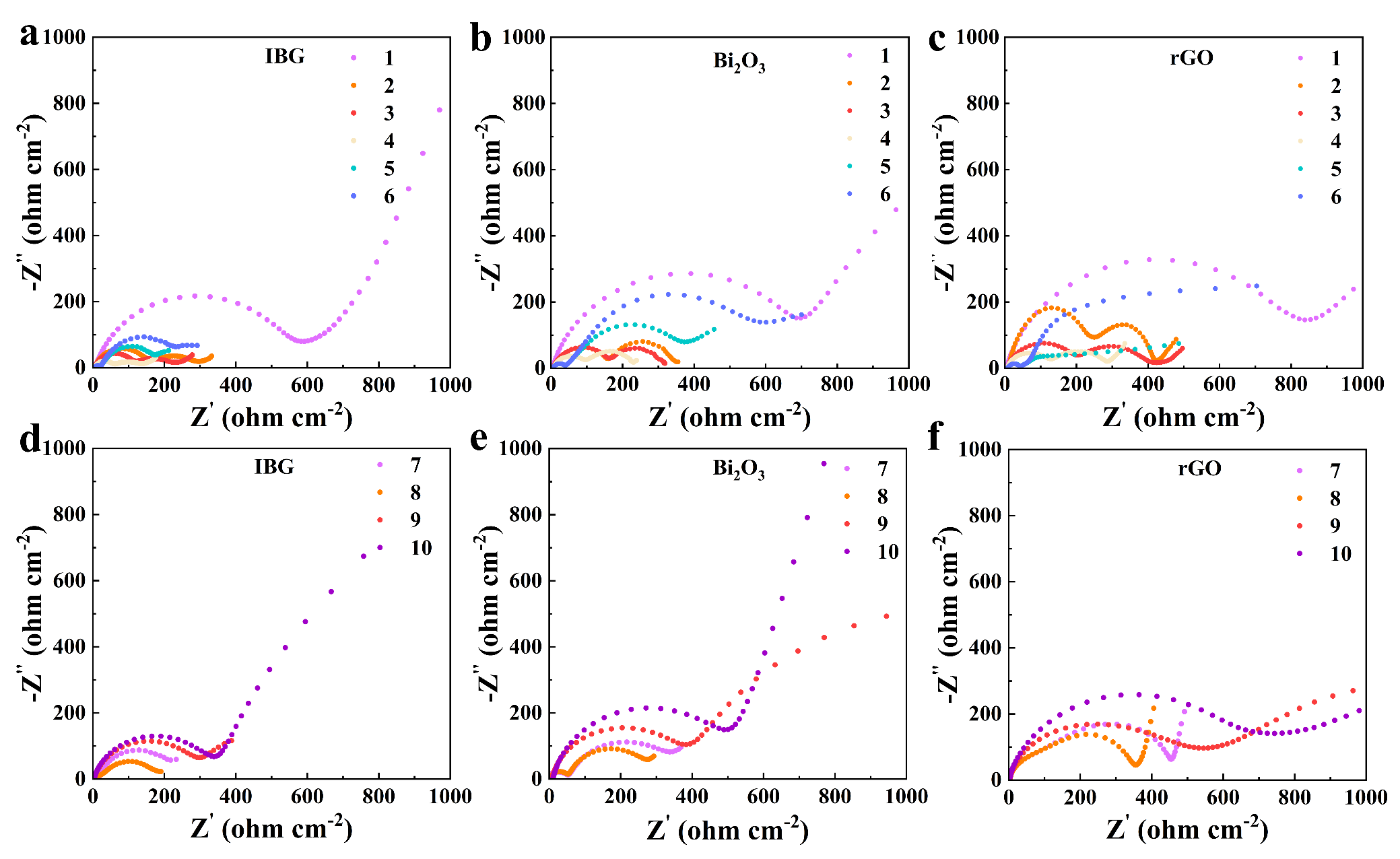


**Figure S6.** Nyquist plot of three electrode materials (IBG, Bi_2_O_3_, rGO) at different stages of initial charge and discharge


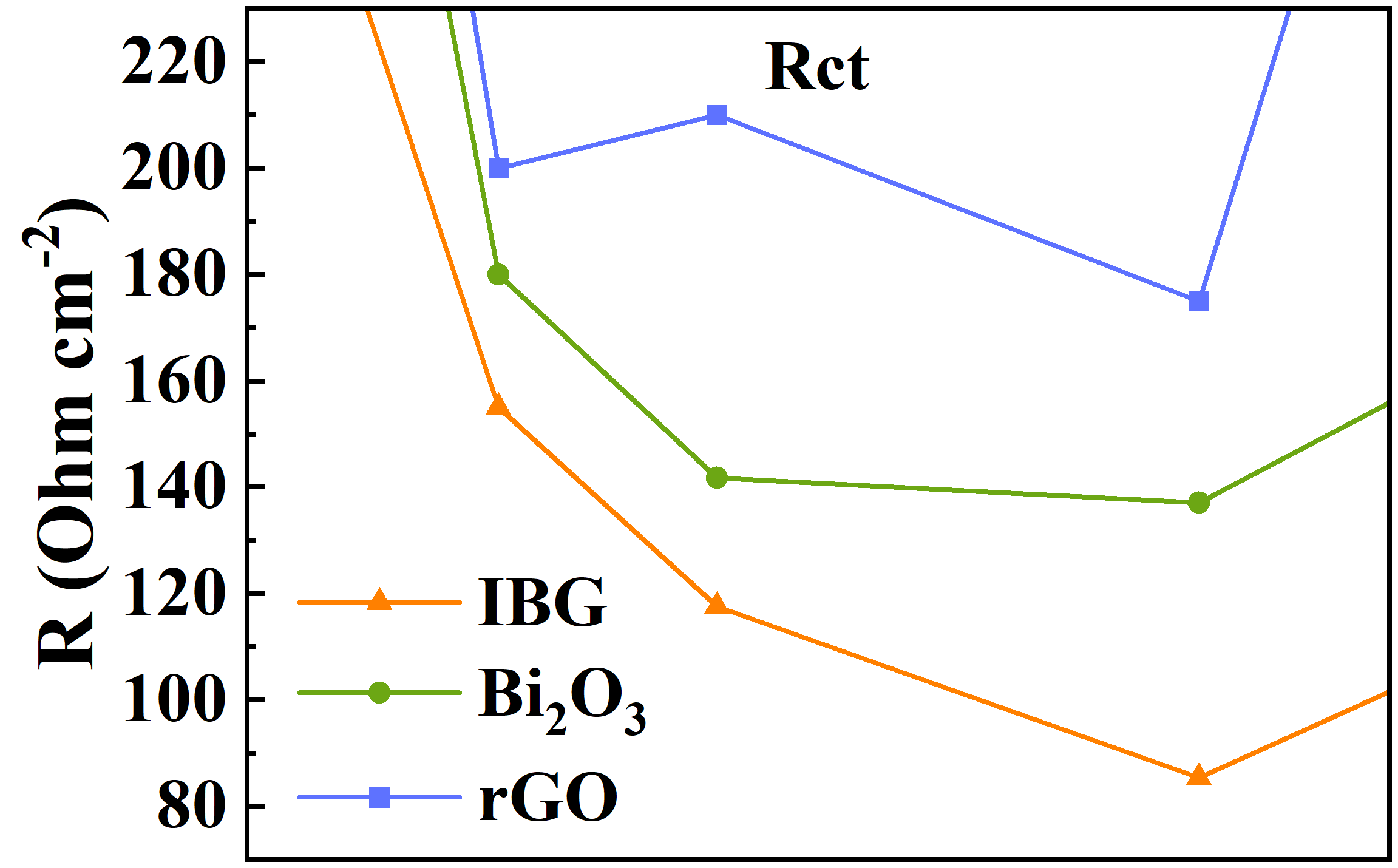


**Figure S7.** Evolution of the different resistance *R_ct_* at -20°C values of cell with IBG, Bi_2_O_3_ and rGO cells.


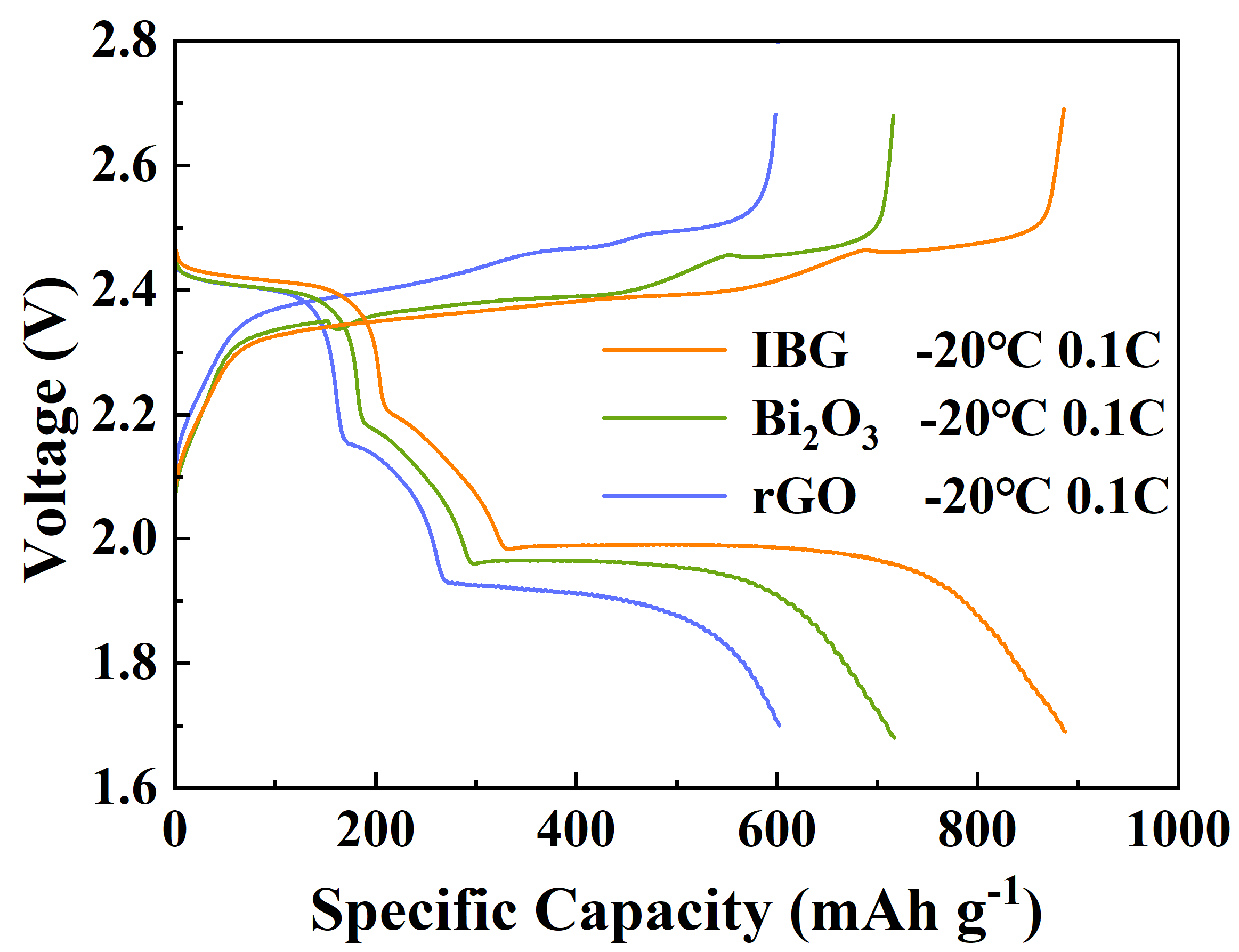


**Figure S8.** Charge and discharge curves of IBG, Bi_2_O_3_, rGO cathodes at 0.1 C at -20℃.


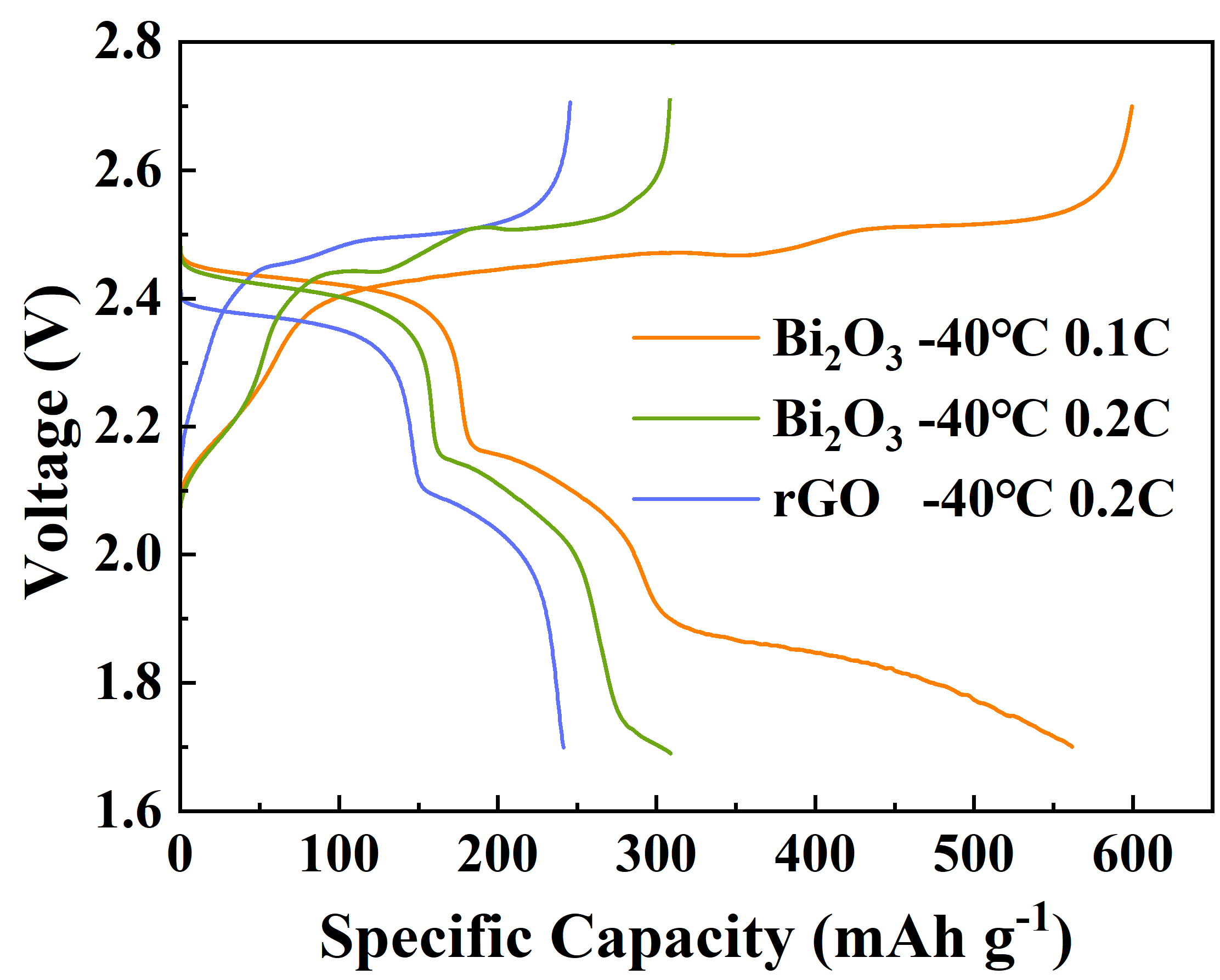


**Figure S9.** Charge and discharge curve of Bi_2_O_3_ at 0.1 C and 0.2 C and charge and discharge curve of rGO cathode at 0.2 C at -20℃


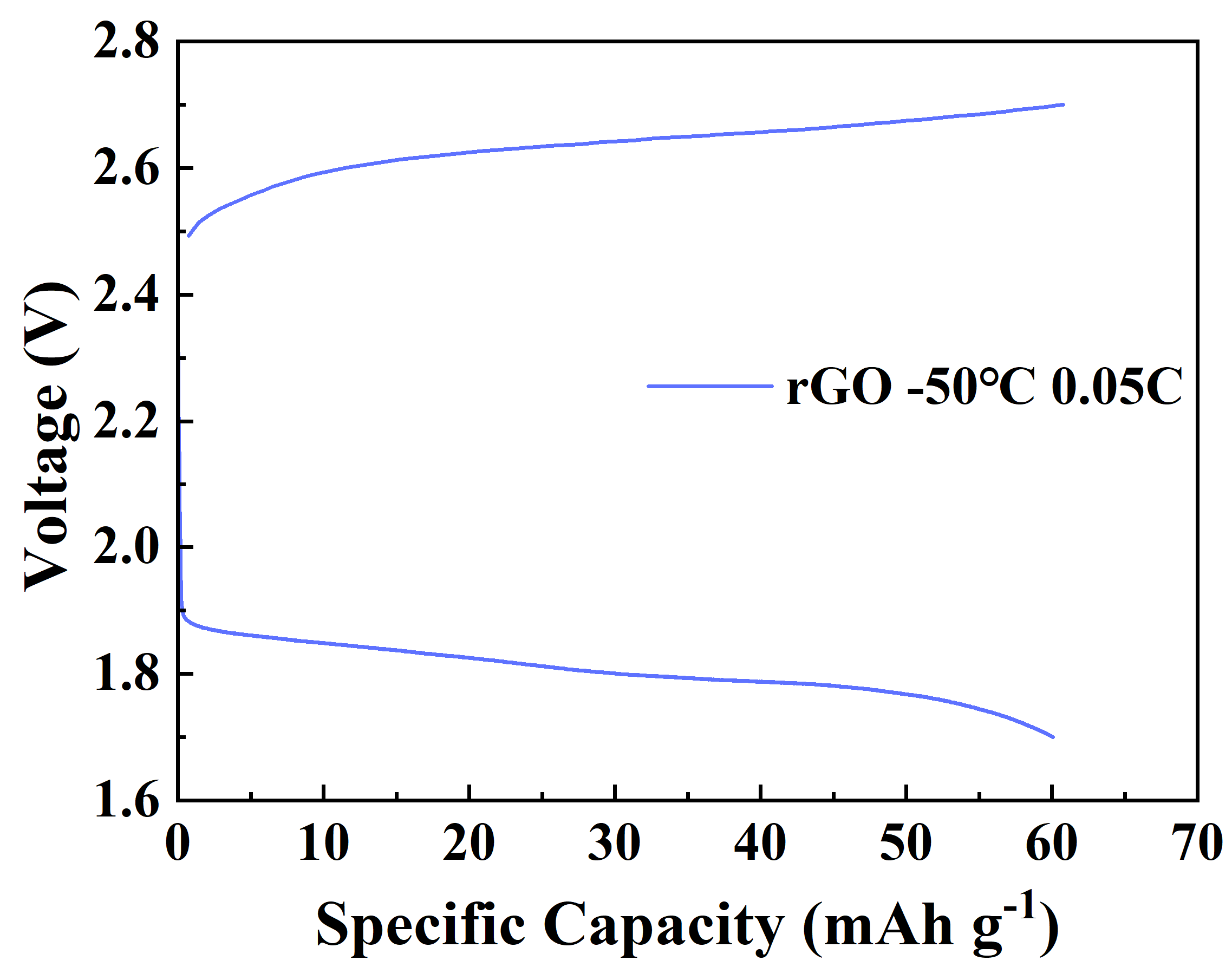


**Figure S10.** Charge and discharge curve of rGO at 0.05 C at -50℃.

**Table S1.** Adsorption Energies of Li_2_Sn at different locations/samples.

| Adsorption energy (eV) | S_8_ | Li_2_S_8_ | Li_2_S_6_ | Li_2_S_6_ | Li_2_S_2_ | Li_2_S |
| --- | --- | --- | --- | --- | --- | --- |
| rGO | -0.52 | -0.68 | -0.57 | -0.52 | -0.78 | -0.79 |
| Bi_2_O_3_ | -0.75 | -2.41 | -2.41 | -2.39 | -2.87 | -3.15 |

**Table S2.** Lithium ion diffusion coefficient D_Li+_ (cm^2^ s^-1^) of peak A, B, C and D.

| peak | D_Li_^+^(cm^2^ s^-1^) of  S/rGO | D_Li_^+^(cm^2^ s^-1^) of  S/Bi_2_O_3_ | D_Li_^+^(cm^2^ s^-1^) of  S/IBG |
| --- | --- | --- | --- |
| A | (1.13 ± 0.01) × 10^-8^ | (4.01 ± 0.01) × 10^-8^ | (9.43 ± 0.01) × 10^-8^ |
| B | (2.45 ± 0.01) × 10^-9^ | (1.37 ± 0.01) ×10^-8^ | (1.76 ± 0.01) × 10^-8^ |
| C | (1.13 ± 0.01) × 10^-9^ | (1.73 ± 0.01) × 10^-9^ | (2.45 ± 0.01) × 10^-9^ |
| D |  | (1.01 ± 0.01) × 10^-9^ | (5.97± 0.01) × 10^-9^ |

**Table S3.** Non-dimension expressions for the SH and BFT nucleation models.

| SH model | |
| --- | --- |
| (3DI) | $\frac{I^{2}}{I_{m}^{2}}=\frac{1.9542}{\frac{t}{t_{m}}}\left\{ 1-exp\left[ -1.2564\left( \frac{t}{t_{m}} \right) \right] \right\}^{2}$ |
| (3DP) | $\frac{I^{2}}{I_{m}^{2}}=\frac{1.2254}{\frac{t}{t_{m}}}\left\{ 1-exp\left[ -2.3367\left( \frac{t}{t_{m}} \right)^{2} \right] \right\}^{2}$ |
| BFT model | |
| (2DI) | $\frac{I}{I_{m}}=\frac{t}{t_{m}}exp\left\{ \frac{1}{2}\left[ 1-\left( \frac{t}{t_{m}} \right)^{2} \right] \right\}$ |
| (2DP) | $\frac{I}{I_{m}}=\left( \frac{t}{t_{m}} \right)^{2}exp\left\{ \frac{2}{3}\left[ 1-\left( \frac{t}{t_{m}} \right)^{3} \right] \right\}$ |

**Table S4.** Electrochemical performance of the various reported LSBs cathodes and present work at low temperatures.

| **S cathode** | **Tempe-rature** | **Rate** | **Initial Capacity (mAh g^-1^)** | **Cycle Number** | **Capacity (mAh g^-1^)** | **References** |
| --- | --- | --- | --- | --- | --- | --- |
| (NH_4_)_2_Mo_2_S_12_ | -20°C | 1C | 280 | 400 | 194 | Ref. 27 |
| TiO_2_@C@CSC | -40°C | 0.2C | 650 | 200 | 350 | Ref. 36 |
| CoFe@C@CNFs | -20°C | 0.2C | 830 | 100 | 790 | Ref. S1 |
| In_2_O_3_@S@C | -10°C | 0.2C | 750 | 400 | 500 | Ref. S2 |
| Cu-g-C_3_N_4_ | 0°C | 0.5C | 995 | 60 | 794 | Ref. S3 |
| In_2_O_3_@NC-Co_3_O_4_ | -20°C | 0.1C | 754 | 100 | 721 | Ref. S4 |
| MnO NPs | -40°C | 1C | 360 | 210 | 109 | Ref. S5 |
| IBG | -20°C | 0.1C | 887 |  |  | This work |
|  |  | 1C | 570 | 800 | 480 |  |
|  | -40°C | 0.1C | 690 |  |  |  |
|  |  | 0.2C | 450 | 100 | 420 |  |
|  | -50°C | 0.05C | 599 | 50 | 530 |  |
|  | -60°C | 0.05C | 350 |  |  |  |

References

1. N. Gao, Y. J. Z, C. Chen, B. Li, W. B. Li, H. Q. Lu, L. Yu, S. M. Zheng, B. Wang, *J. Mater. Chem. A* **2022**, *10*, 8378-8389.
2. J, Liu. Y. Ding. Z. Shen, H. Zhang, T. Han, Y. Guan, P. V. Braun, *Adv. Sci.* **2022**, *9*, 2103517.
3. W. Q. Zhang, M. F. Chen, Y. X. Luo, Y. Q. He, S. S. Liu, Y. J. Ye, M. Q. Wang, Y. Chen, K. Zhu, H. B. Shu, M. Liu, J. H. Hou, T. F. Duan, X. Y. Wang, *Chem. Eng. J.* **2024**, *486*, 150411.
4. T. C. Wang, F. R. Wang, Z. H. Shi, S. R. Cui, Z. Q. Zhang, W. Liu, Y. C. Jin, Available at *SSRN* 4681255.
5. X. W. Pang, B. G. An, S. M. Zheng, B. Wang, *Chem. Eng. J.* **2023**, *458*, 141445.
